# Small extracellular vesicles and their miRNA cargo are anti-apoptotic members of the senescence-associated secretory phenotype

**DOI:** 10.18632/aging.101452

**Published:** 2018-05-19

**Authors:** Lucia Terlecki-Zaniewicz, Ingo Lämmermann, Julie Latreille, Madhusudhan Reddy Bobbili, Vera Pils, Markus Schosserer, Regina Weinmüllner, Hanna Dellago, Susanna Skalicky, Dietmar Pum, Juan Carlos Higareda Almaraz, Marcel Scheideler, Frédérique Morizot, Matthias Hackl, Florian Gruber, Johannes Grillari

**Affiliations:** 1 Christian Doppler Laboratory for Biotechnology of Skin Aging, Vienna, Austria; 2 Department of Biotechnology, University of Natural Resources and Life Sciences, Vienna, Austria; 3 Department of Biology and Women Beauty, Chanel R&T, Pantin, France; 4 Division for Biology and Pathobiology of the Skin, Department of Dermatology, Medical University of Vienna, Vienna, Austria; 5 Institute for Diabetes and Cancer (IDC), Helmholtz Zentrum München, German Research Center for Environmental Health, Neuherberg, Germany; 6 Joint Heidelberg-IDC Translational Diabetes Program, Heidelberg University Hospital, Heidelberg, Germany; 7 Molecular Metabolic Control, Medical Faculty, Technical University Munich, Germany; 8 German Center for Diabetes Research (DZD), Neuherberg, Germany; 9 TAmiRNA GmbH, Vienna, Austria; 10 Department of Nanobiotechnology, University of Natural Resources and Life Sciences, Vienna, Austria

**Keywords:** senescence-associated secretory phenotype (SASP), cellular senescence, small extracellular vesicle (sEV), exosomes, microRNA (miRNA)

## Abstract

Loss of functionality during aging of cells and organisms is caused and accompanied by altered cell-to-cell communication and signalling. One factor thereby is the chronic accumulation of senescent cells and the concomitant senescence-associated secretory phenotype (SASP) that contributes to microenvironment remodelling and a pro-inflammatory status. While protein based SASP factors have been well characterized, little is known about small extracellular vesicles (sEVs) and their miRNA cargo. Therefore, we analysed secretion of sEVs from senescent human dermal fibroblasts and catalogued the therein contained miRNAs. We observed a four-fold increase of sEVs, with a concomitant increase of >80% of all cargo miRNAs. The most abundantly secreted miRNAs were predicted to collectively target mRNAs of pro-apoptotic proteins, and indeed, senescent cell derived sEVs exerted anti-apoptotic activity. In addition, we identified senescence-specific differences in miRNA composition of sEVs, with an increase of miR-23a-5p and miR-137 and a decrease of miR-625-3p, miR-766-3p, miR-199b-5p, miR-381-3p, miR-17-3p. By correlating intracellular and sEV-miRNAs, we identified miRNAs selectively retained in senescent cells (miR-21-3p and miR-17-3p) or packaged specifically into senescent cell derived sEVs (miR-15b-5p and miR-30a-3p). Therefore, we suggest sEVs and their miRNA cargo to be novel, members of the SASP that are selectively secreted or retained in cellular senescence.

## Introduction

Accumulation of senescent cells with age and at sites of age-associated diseases has been observed in the context of cardiovascular diseases, neurodegenerative disease, skin conditions and others [1]. Importantly, their removal in transgenic mice [2–4] or by senolytics [5,6] leads to later onset of several age-associated diseases [2,7–9].

Cellular senescence is triggered by various stimuli such as progressive telomere-shortening, hyperoncogenic signaling, accumulation of DNA damage, oxidative stress or mitochondrial dysfunctions, leading to an irreversible growth arrest mediated by the key cell cycle inhibitors CDKN1A and/or CDKN2A [10]. Most cell types activate pro-survival pathways and resist apoptosis when senescent [11]. They lose their cell type specific functionality and replicative potential required for tissue regeneration and acquire a senescence-associated secretory phenotype (SASP) [12].

The SASP is characterized by the secretion of growth factors, pro-inflammatory cytokines and chemokines, as well as extracellular matrix (ECM) remodeling enzymes [12]. These SASP factors are considered to over-proportionally exert negative effects on tissue homeostasis and regeneration *in vivo* if chronically present by acting in a paracrine manner on the neighboring cells and ECM. Attenuation of the negative effects of the SASP have been shown to restore the formation of functional human skin equivalents [13] and has been suggested as a putative target in preventing age-associated diseases and frailty [8,14].

Recently, extracellular vesicles (EVs) and their cargo have been reported to act in a similar manner as hormones or cytokines during intercellular communication [15]. They are secreted by many, if not all cells, and by encapsulation of their cargo, they transport proteins, mRNAs, lipids and non-coding RNAs, specifically miRNAs, over short or long distances [16]. When taken up by recipient cells, the cargo is considered to be still active and to regulate the behavior of recipient cells [17,18].

MiRNAs clearly modulate cellular senescence and organismal aging *in vitro and vivo* [19,20] and are in addition packaged into EVs [21], where they are able to influence osteogenic differentiation as one major age-associated disease [22]. Thus, although many protein based SASP factors have been identified, miRNAs [23,24] and EVs [25] are under suspicion to be part of the SASP [26,27]. However, a systematic catalogue of SASP-miRNAs has not yet been established and their selective secretion during senescence has not been studied so far.

Here, we confirm that EVs and their miRNA cargo are indeed part of the SASP (EV-SASP) and identified a set of selectively retained and secreted miRNAs after the onset of senescence. In addition, senescent cell derived EVs might contribute to an anti-apoptotic environment in tissues where senescent cells have accumulated.

## RESULTS

### sEVs are members of the senescent-associated secretory phenotype (EV-SASP)

In order to test whether EVs and their enclosed miRNAs are members of the SASP, primary human dermal fibroblasts (HDF) of three different donors were driven into premature senescence by exposing them repeatedly to hydrogen peroxide (H_2_O_2_) [28]. Analysis were then performed one (D7) and 3 weeks (D21) after the last H_2_O_2_ treatment (Fig. S1A).

**Figure 1:**
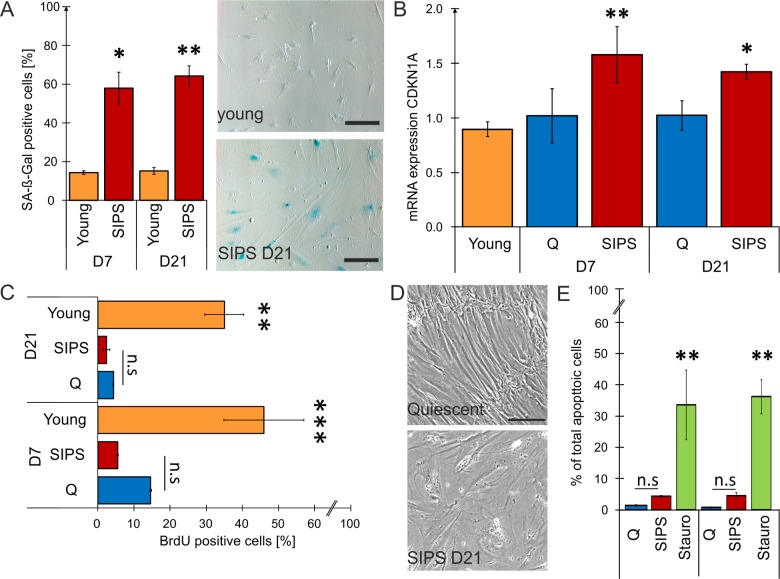
**Stress-induced premature senescent (SIPS) fibroblasts mirror hallmarks of cellular senescence.** (**A**) Quantification of SA-ß-Gal staining shows a significant increase of ß-Gal in SIPS HDF compared to young proliferating cells at both time points post stress treatment. Representative pictures show SA-ß-Gal staining of donor HDF161 in SIPS on D21 (bottom) compared to young proliferating control (top - HDF161 in population doublings PD15). 15 pictures were taken randomly at a magnification of 100 X and counting was performed in a blinded fashion. Scale bar = 200 µm. Percentages of SA-ß-Gal positive cells from all pictures were calculated. (**B**) Expression of CDKN1A confirms senescence of SIPS HDF at both time points. mRNA expression levels of CDKN1A (p21) were detected by qPCR. After normalization to GAPDH, fold changes of SIPS HDF relative to quiescent (Q) control cells from D7 were calculated. (**C**) SIPS treatment induces permanent cell cycle arrest. Incubation with the nucleoside derivate BrdU for 24 hours followed by FITC immunolabelling for flow cytometry shows no significant incorporation of BrdU into the DNA of Q and SIPS samples compared to young dividing HDF at both time points. (**D**) SIPS cells show flattened and enlarged morphology. Representative pictures from donor HDF161 Q and SIPS on D21 post H_2_O_2_ treatment. Scale bar = 200 µm. (**E**) Repeated H_2_O_2_ treatment does not induce apoptosis. SIPS and Q control cells do not show a substantial increase in percentage (%) of total apoptotic cells at both time points compared to a positive-control (+), treated with 300 nM staurosporin for 24 hours. (**A**-**E**) Stress-induced premature senescence (SIPS) of primary human dermal fibroblasts (HDF) derived from three different donors was triggered by chronic H_2_O_2_ treatment on nine consecutive days. Hallmarks of cellular senescence were confirmed after seven (D7) and 21 days (D21) post last stress treatment. Averages from three biological triplicates are shown +/- SEM from raw values (n = 3). Statistical analysis was performed using 2-way RM ANOVA tested for condition and day following Bonferroni post test. n.s ≥ 0.05; *p < 0.05; **p < 0.01.

Onset and persistence of cellular senescence was confirmed in detail day 7 and 21 (Table 1A and B), by senescence-associated (SA)-ß-Gal staining (Fig. 1A), expression of CDKN1A/p21 (Fig. 1B), induction of an irreversible growth arrest (Fig. 1C), as well as by the acquiring of a fibroblast-specific flattened and enlarged senescent phenotype (Fig. 1D). In order to exclude contamination of EV preparations by apoptotic bodies, basal apoptosis rates of quiescent (Q) and senescent cells (SIPS) were analysed, whereby no significant difference was detected (Fig. 1E) at time points of EV purification as outlined in the scheme of Fig. S1A.

**Table 1A:** Detailed characteristics of major hallmarks of cellular senescence of the individual donors HDF161, HDF76 and HDF85 each senescent and quiescent control after 7 (D7) days post last H_2_O_2_ application.

**1 week recovery (D7)**													
**Donor**	**Total apoptotic cells (Annexin + PI positive) [%]**			**BrdU positive cells [%]**			**Sub-G1 peak (PI staining) [%]**		**CDKN1A mRNA expression norm.FC**			**SA-βGal positive cells [%]**	
	**Q**	**S**	**+**	**Q**	**S**	**Y**	**Q**	**S**	**Q**	**S**	**Y**	**Y**	**S**
**HDF161**	**1.7**	**4.3**	**49**	**15**	**5**	**55**	**2.5**	**3.9**	**0.6**	**1.1**	**0.9**	**16.1**	**50**
**HDF85**	**1.1**	**4.1**	**12**	**14**	**2.8**	**35**	**0.7**	**4.8**	**1.3**	**1.7**	**1.0**	**13**	**74**
**HDF76**	**1.5**	**4.7**	**40**	**15**	**8**	**48**	**1.2**	**3.3**	**1.2**	**1.9**	**0.8**	**13.8**	**49**

Abbreviations: Q = quiescent control, S = stress induced premature senescent, + = positive control – Staurosporin 300nM overnight, Y = proliferating control.

**Table 1B:** Detailed characteristics of major hallmarks of cellular senescence of the individual donors HDF161, HDF76 and HDF85 each senescent and quiescent control after 21 days (D21) post last H_2_O_2_ application.

**3 week recovery (D21)**												
**Donor**	**Total apoptotic cells (Annexin + PI positive) [%]**			**BrdU positive cells [%]**			**Sub-G1 peak (PI staining) [%]**		**CDKN1A mRNA expression norm.FC**		**SA-βGal positive cells [%]**	
	**Q**	**S**	**+**	**Q**	**S**	**Y**	**Q**	**S**	**Q**	**S**	**Y**	**S**
**HDF161**	**1.1**	**2.8**	**40**	**4.5**	**1.5**	**35**	**0.8**	**3.3**	**1.2**	**1.3**	**13**	**70**
**HDF85**	**0.7**	**5.4**	**26**	**3.8**	**0.4**	**16**	**1.2**	**3.2**	**1.1**	**1.5**	**14**	**69**
**HDF76**	**0.6**	**5.6**	**43**	**3.5**	**1.1**	**54**	**1.3**	**6.8**	**0.8**	**1.4**	**19**	**54**

Abbreviations: Q = quiescent control, S = stress induced premature senescent, + = positive control – Staurosporin 300nM overnight, Y = proliferating control.

**Figure 2:**
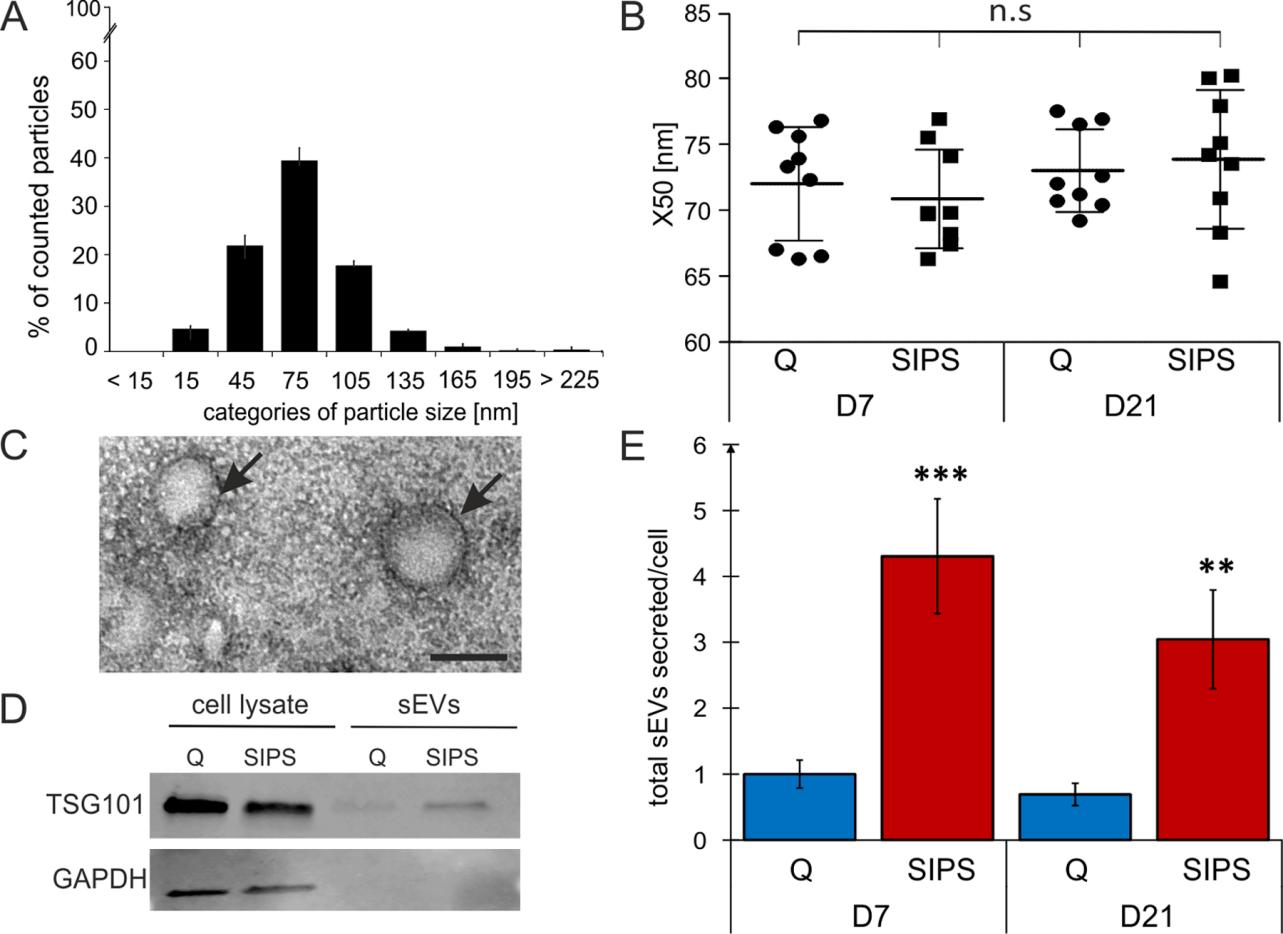
**sEVs are members of the senescent-associated secretory phenotype (EV-SASP).** (**A**) NTA reveals a vesicle population below 220 nm. Size distribution of vesicles determined by NTA shows percentage (%) of total counted particles against size presented in categories. (**B**) Media values (X50) from sEVs range from 65 to 80 nm. X50 values from peak analysis of NTA are indicated +/- SEM. circle: Q, squares: SIPS. Statistical analysis using one-way ANOVA was performed: not significant (n.s) p > 0.05. (**C**) Representative transmission electron microscopy image of sEVs isolated from HDF. Vesicles are around 100 nm in size and are surrounded by a double lipid membrane (arrows). Scale bar = 100 nm. A representative image of sEVs purified from HDF85 at D7 after the stress treatment is shown. (**D**) Representative Western blot shows expression of TSG101 (top) and GAPDH (below). Representative Western blot of total cell lysates (left) and sEVs (right lanes) from Q and SIPS HDF of donor HDF85 are shown. Total protein content of total cell lysates and purified sEV was analyzed by BCA assay and equal amounts of protein were loaded onto the gel (20 µg). (**E**) Senescent cells secrete more sEVs per cell than quiescent controls. Total concentration of tracked particles was normalized to the total cell number used for secretion into conditioned media. Fold changes of total particles secreted per cell, relative to Q control cells from D7, +/- relative SEM, are shown. Statistical analysis was performed using 2-way RM ANOVA tested for condition (p < 0.0001) and day (p = 0.28) following Bonferroni post test. **p < 0.01; ***p < 0.01. (**A**-**B** and **E**) Averages from three biological triplicates (n = 3) and two different time points each SIPS and Q, were measured in technical triplicates (n = 18) +/- relative SEM.

We here focused on small EVs (sEVs), therefore supernatants of SIPS and Q control cells were filtrated using 0.22 µm filters and subsequently ultracentrifugated. Size distribution as assessed by nanoparticle tracking analysis (NTA) revealed sEVs between 15 to 135 nm (Fig. 2A) with a median diameter of 65 to 80 nm, with no difference between senescent and quiescent cells at both time points, 7 and 21 days after the last stress treatment (Fig. 2B). Transmission electron microscopy showed typical morphology and presence of lipid bilayers (Fig. 2C), and Western blotting confirmed the presence of TSG101, a known marker for exosome-like vesicles (Fig. 2D).

Finally, we compared the number of sEVs per cell by NTA and observed a 4-fold increased secretion of senescent fibroblasts derived sEVs of all three donors after both time points of cellular senescence (D7 and D21) (Fig. 2E).

Considering the phenomenon of increased senescence-associated secretion of proteins summarized under the term SASP, our data strongly support the idea that sEVs are members of the SASP, for which we propose the term ‘EV-SASP’.

### sEV-miRNAs as part of the SASP are identified in a preliminary and final qPCR screening

**Figure 3:**
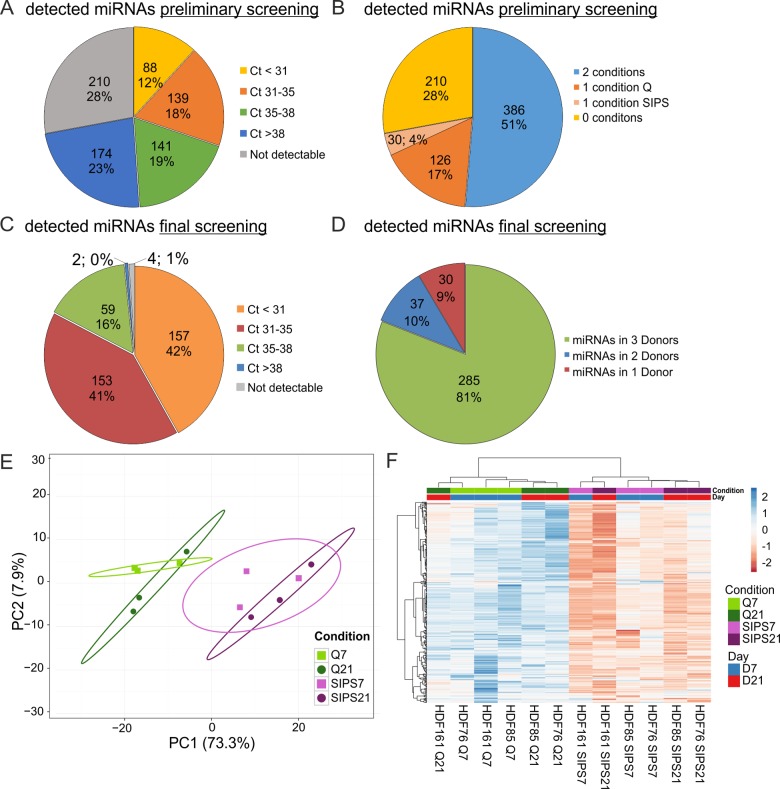
**sEV-miRNAs as part of the SASP were identified in a preliminary and final qPCR screening.** (**A**) miRNA profiling of the preliminary screening detects in total 542 (72%) secreted miRNAs. Categorization of Ct-values shows 368 miRNAs with an average signal < 38 in one or both conditions (Q, SIPS) tested. (**B**) The preliminary screening detects in total 386 miRNAs in both conditions tested. (**C**) The final qPCR screening detects 369 miRNAs with Ct-values below 38. 375 miRNAs were tested in all conditions and time points. % and number of total miRNAs detected in the screening experiment are shown. Categorization according to Ct-values. MiRNAs with an average Ct-value < 31, between 31 and 35, between 35 and 38, > 38 and not detectable are displayed. (**D**) The final qPCR screening detects 81% of all screened miRNAs in three donors. Averages from D7 and D21 are presented. 81% (285) of miRNAs were detected in all three donors SIPS and Q. 10% (37) of miRNAs were detected in at least two donors and 9% (30) of miRNAs were detected in one donor. (**E**) Principal Component analysis of sEV-miRNAs from SIPS and Q control cells from day 7 (D7) and day 21 (D21) after the treatment. The expression matrix shows the clustering of 12 samples and 369 miRNAs. Ellipses indicate a confidence level of 95% that a new observation will fall into it. Illustrated 2D-biplot explains a variance of 73.3% in principal component 1 and 7.9% in principal component 2, respectively. Exploratory analysis was done with ClustVis. *Green*: Q; *Purple*: SIPS; *light colors and rectangular* D7; *dark colors and circle* D21. (**F**) sEV-miRNAs are higher secreted from SIPS cells compared to Q controls. Heatmap and hierarchical clustering of 369 sEV-miRNAs after D7 and D21 (n = 12). Unit variance scaling was applied and rows are centered. MiRNAs were clustered according to correlation distance and Ward linkage method. Samples in columns are clustered using Euclidean distance and Ward linkage method. *Green*: Q; *Purple*: SIPS; *light colors and blue* D7; *dark colors and red* D21. Colors in matrix: red = upregulated, blue = downregulated. (**A**-**B**) Magnitude of secreted sEV-miRNAs was assessed in a preliminary screening using Q control and SIPS HDF of one cell strain (HDF76) and from one time point (D21). 752 miRNAs were screened using the qPCR ready to use panels supplied by Exiqon. (**C**-**F**) Final screening was performed with customized qPCR panels using three different HDF cell strains (n = 3) each Q and SIPS from two different time points (D7 and D21).

In order to determine which miRNAs are detectable in sEVs from quiescent control and senescent cells, a preliminary screening using a qPCR-panel of 752 miRNAs was performed and analysed in detail (Fig. 3A-B). From these, we designed a customized (final) qPCR panel with 375 miRNAs and spike in-controls (Supplementary List S1). Within that, 369 miRNAs were detected at Ct-values ≤ 38 (Fig. 3C) and 285 miRNAs were found in all three HDF cell strains under both conditions and at both time points (Fig. 3D).

As quality control, interplate variation and PCR efficiency was monitored using five synthetic spike-ins (Unisp2, Unisp4, Unisp5, Unisp6, cel-miR39) controlling for RNA extraction, cDNA synthesis, and qPCR efficiency, resulting in ΔCt_r_-values (range of highest and lowest Ct-value from all samples each) below 1. Additionally, each plate included two interplate calibrators (IPC) and a negative control, showing ΔCt_r_-values below 0.44 suggesting robust signals (Fig. S2A) and thus allowing to exclude inter-assay variations.

Due to the absence of a robust extracellular housekeeping miRNA, we used standardized secretion times and volumes for vesicle preparations and subsequent RNA isolation and normalized the data to the total viable cell number of each sample (Table 2).

**Table 2:** Summary and evaluation of secreted miRNAs by qPCR panels.

	**Preliminary screening**		**Final screening**	
**conditions**	**Q**	**SIPS**	**Q**	**SIPS**
biological replicates (HDF)	N = 1	N = 1	N = 3	N = 3
Time points (days post treatment)	D21	D21	D7; D21	D7; D21
number of cells used for sEV-RNA (average)	1.27E+07	9.13E+05	1,50E+07 ± 33%	1,65E+06 ± 30%
screened miRNAs (Exiqon)	752		375	
detected miRNAs in 2 conditions (average)	386		371	
detected miRNAs in 1 condition (average)	156		0	
not detected (average)	210		4	
**raw Ct-values normalized to number of cells used for sEV-RNA**				
detected miRNAs Ct_(Average)_ ≤ 31	101	187	112	220
detected miRNAs Ct_(Average)_ 31 - 35	197	142	138	132
detected miRNAs Ct_(Average)_ 35 - 38	459	313	121	19
detected miRNAs Ct_(Average)_ > 38	366	235	0	0
**Dataset of miRNAs for statistic quantification (Ct_(Average)_ < 38)**			353	
miRNAs with complete dataset for 3 Donor D7/D21			280/290	
miRNAs with complete dataset for 2 Donor D7/D21			36/38	
miRNAs with complete dataset for 1 Donor D7/D21			36/24	

Preliminary screening of secreted miRNAs to determine detectable miRNAs in small EVs derived from HDFs, was performed using one HDF strain in both conditions, stress-induced premature senescent (SIPS) and quiescent control (Q), from one time point (D21). 375 miRNAs out of 752 screened were selected for the final screening with three different HDF cell strains (n = 3), in 2 conditions (SIPS and Q) and from two time points at 7 (D7) and 21 days (D21) after treatment.

Multivariate statistics on the 369 sEV-miRNAs clearly distinguished senescent from quiescent control cells as depicted by principal component analysis (Fig. 3E) and hierarchical clustering (Fig. 3F), showing an increase of almost all sEV-miRNAs. Due to 4-fold more sEVs per cell it is of no surprise, that almost all miRNAs are upregulated in the supernatants of senescent cells as indicated by the heatmap (Fig. 3F). Indeed, statistical evaluation confirmed 221 miRNAs (59%) with significantly higher secretion levels on D7 (Fig. S2B), whereby miR-200c-3p and miR-196b-3p were identified to be the most differentially secreted miRNAs per cell. 3 weeks after induction of senescence (D21), 321 (85%) miRNAs were confirmed to be differentially secreted and miR-23a-5p reached the highest level (Fig. S2C), while none were downregulated significantly at both time points (Supplementary List S2). Thus, our findings indicate that sEVs and their miRNA cargo are *bona fide* members of the SASP.

### Senescent cell derived sEVs confer anti-apoptotic activity

**Figure 4:**
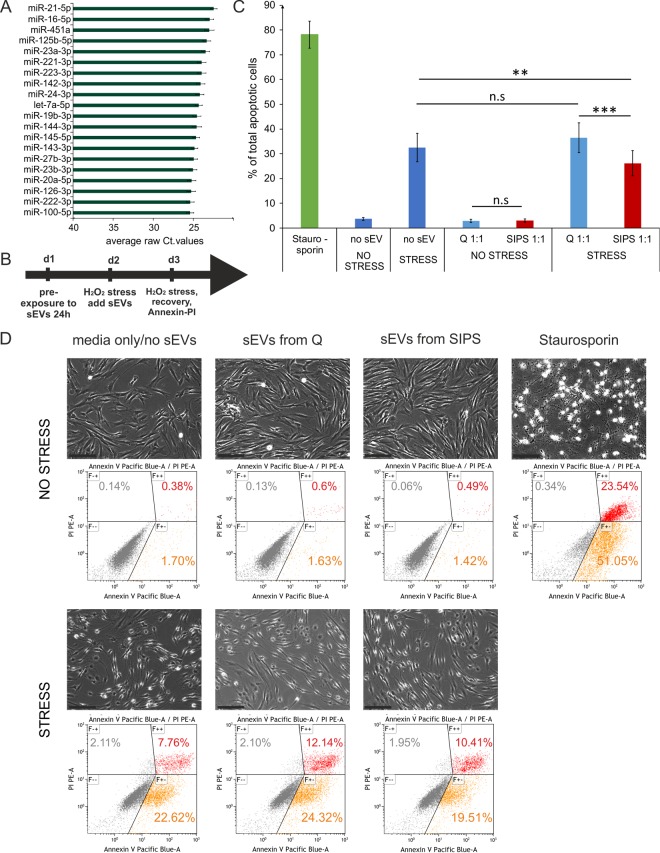
**Senescent cell derived sEVs confer anti-apoptotic activity.** (**A**) Barchart of the top 20 most highly secreted sEV-miRNAs. To cell count normalized Ct-values from Q and SIPS from two time points were averaged and are plotted +/- SEM derived from all 12 samples. (**B**) Experimental setup to test the biological effect of the EV-SASP. Recipient fibroblasts were pre-exposed to sEVs for 24 hours followed by an acute stress treatment for 2 hours with 200 µM H_2_O_2_ and fresh sEVS were added_._ On the next day a second stress treatment with 400 µM for 2 hours was performed followed by a recovery time of 3 hours. Annexin-V-PI staining and flow cytometric measurement was used to determine % total number of apoptotic cells. (**C**) The EV-SASP reduces the amount of apoptotic cells of oxidatively stressed recipient cells. sEVs of SIPS and Q control cells of three different donors between 2 to 4 weeks of recovery post SIPS treatment were freshly harvested and applied before and after acute stress treatments. Human primary dermal (n = 3) and foreskin fibroblasts (n = 3) were used as recipient cells. Averages from 6 independent experiments +/- SEM are shown. Statistical analysis (n = 6) using 2-way RM ANOVA identified the factor 'EV/no EV' as a significant subject (p = 0.014) and the factor 'no stress/stress' as a significant factor (p = 0.00014). Groups were compared by Bonferroni post test, n.s ≥ 0.05; **p < 0.01, ***p < 0.01. (**D**) Representative pictures of recipient fibroblasts of all conditions tested prior Annexin-V-PI staining. Representative flow cytometric data are shown. Scale bar = 200 µm.

In order to get insight into a potential function of the EV-SASP, the top 20 most abundant sEV-miRNAs were identified (Fig. 4A) and screened for validated miRNA/mRNA target pairs. Thereby, we found in total 11,588 interactions comprising 5,437 target genes (Supplementary List S3). To evaluate potential regulated pathways, enrichment analysis of all annotated interactions between miRNAs and genes, discovered 125 GO terms with an adjusted p-value below 0.0001, among those, 54 comprise more than 50% of all associated genes (Fig. S3A).

Interestingly, the top 20 highly secreted miRNAs Fig. 4D) were predicted to regulate a dynamic crosstalk of three prominent meta-pathways by targeting five common transcription factors (PTEN, P53, APAF-1, CDKN1B and MYC) (Fig. S3B) that are also well known pro-apoptotic mediators [11,29–33].

Therefore, acutely stressed recipient fibroblasts were exposed to the entirety of senescent or quiescent cell derived sEVs and Annexin-V-PI staining for assessing apoptosis rates was performed (Scheme Fig. 4B). Indeed, the presence of senescent cell derived sEVs reduced the amount of apoptotic cells by approximately 27% (Fig. 4C-D), suggesting an anti-apoptotic activity of the EV-SASP. Whether and which miRNAs exert this effect will be subject of further studies.

### Changes in miRNA composition of senescent cell derived sEVs

While in total almost all sEV-miRNAs are increasingly secreted when compared to cell numbers, we were interested, if also the miRNA composition of sEVs would change during senescence. Therefore, we performed global mean normalization [34] of all miRNAs assuming that the total amount of miRNAs is unchanged within sEVs irrespective of the condition, since vesicle size (Fig. 2B) and global means of total miRNA content from both time points, each SIPS and Q were similar (Fig. S4A).

**Figure 5:**
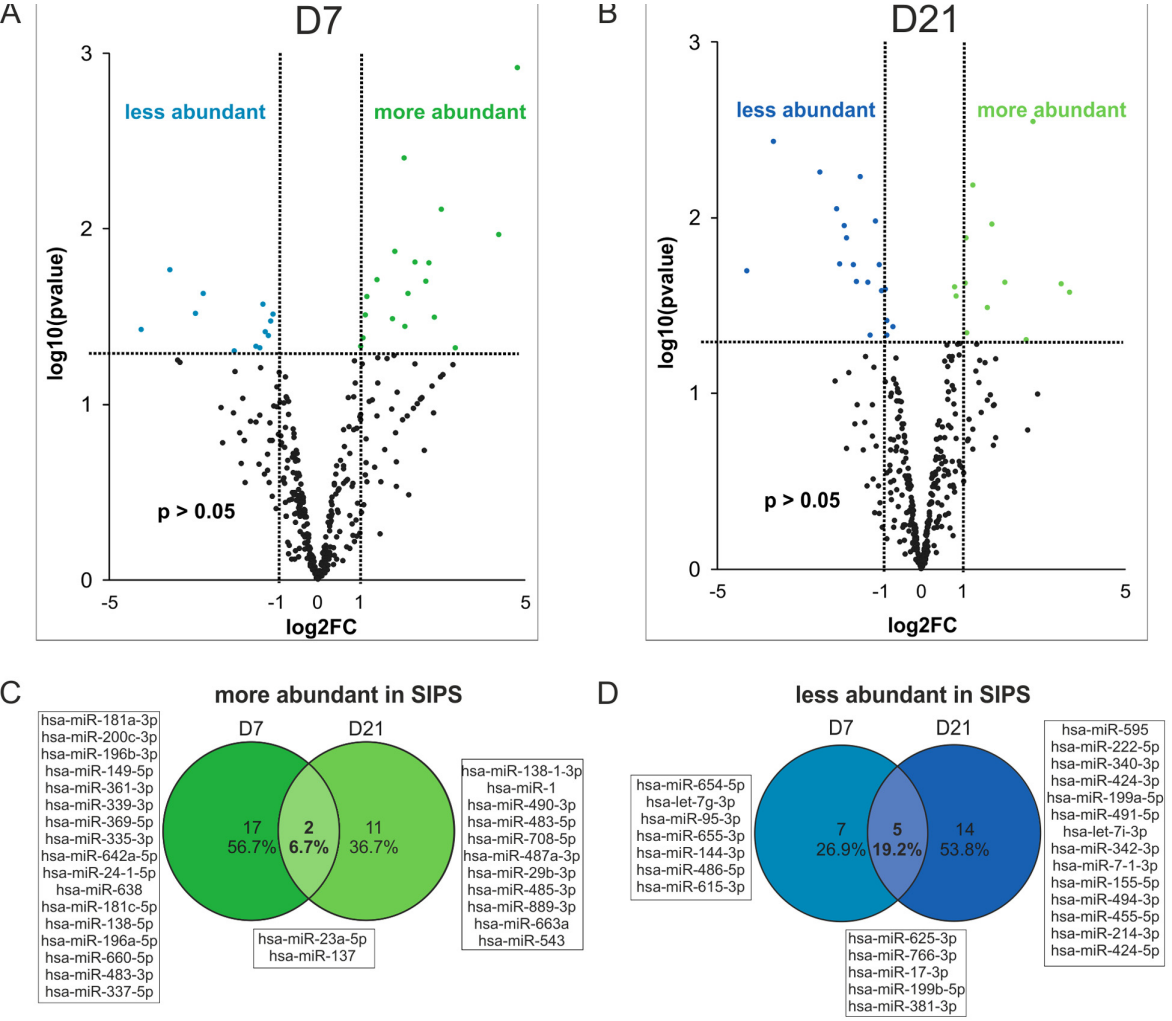
**Changes in miRNA composition of senescent cell derived sEVs.** (**A**) Volcano plot shows 31 significantly differently present senescence-associated (SA) sEV-miRNAs after normalization to the global means at D7 and (**B**) 32 SA sEV-miRNAs at D21 after the last H_2_O_2_ treatment. (**C**) Venn diagram shows miRNAs more abundantly present in sEVs of SIPS cells. (**D**) Venn diagram shows miRNAs less abundant in sEVs of SIPS cells. (**A**-**B**) Raw Ct-values from each sample were normalized to the respective global mean. Log2FC of SIPS relative to Q control cells were calculated. Values from D7 (panel **A**) and D21 (panel **B**) recovery are plotted on x-axis against their individual -log10(p-value) on y-axis. Horizontal dotted lines indicate a separation between miRNAs passing a p-value higher or lower than 0.05. Vertical dotted lines separate secreted miRNAs with log2FC > 1 or log2FC < 1. MiRNAs reaching a p-value < 0.05 are illustrated with green and blue dots and miRNAs with a p-value > 0.05 are shown in black. None reached the 0.05 cut-off value for the FDR of an adjusted p-values. Analysis was performed using three different HDF cell strains (n = 3) each Q and SIPS from two different time points (D7 and D21). (**C**-**D**) Log2FC was calculated and significantly regulated (p-value < 0.05) miRNAs from D7 and D21 were compared in a Venn diagram.

Indeed, statistical analysis identified 31 miRNAs differentially present per sEV at day 7 after induction of cellular senescence (Fig. 5A), and 32 miRNAs at day 21 (Fig. 5B).

Surprisingly, out of these, only two miRNAs (miR-23a-5p, miR-137) were more abundant in sEVs at both time points (Fig. 5C), while five miRNAs (miR-17-3p, miR-625-3p, miR-766-3p, miR-199b-5p, miR-381-3p) were less abundant in sEVs of senescent cells (Fig. 5D).

Taken together, these results indicate that senescent cells do not only secrete more miRNA containing sEVs as part of the SASP, but that in addition the miRNA composition of single sEVs changes with senescence.

### Intracellular miRNA analysis by next generation sequencing (NGS) identifies early and deep senescence specific miRNAs

Since differential secretion of sEV-miRNAs might be caused either by differential transcription, processing or packaging into sEVs, we decided to quantify also the intracellular miRNA composition of all three fibroblast cell strains at both time points (D7 and D21) by small RNA-NGS.

Quality control and results of cDNA library preparation and NGS were assessed (Fig. S5A-F). On average 17.6 million reads per sample were obtained (Fig. S5G) and miRNAs were identified according to miRBase 20.0. The dataset was evaluated (Table 3), normalized to the total number of reads and 432 miRNAs that reached at least five tags per million (TPM) in at least one donor were included into the analysis.

**Table 3:** Summary of miRNA next generation sequencing (NGS) and data quality control.

**Experimental Design**	
Instrument	NextSeq 500
Average number of reads (1 flowcell)	4.00E+08
Number of sequencing cycles	50 bp single-end read
Annotation reference	miRBase 20
**Quality control**	
Base call accuracy (Q-Score)	>30
Averaged Total reads	**1.26E+07 ± 29.38%**
miRNAs (44.2%)	5.64E+06 ± 38.39%
smallRNA (7.8%)	9.78E+05 ± 30.48
Genome-mapped (11.2%)	1.42E+06 ± 35.37%
outmapped (28.5%)	3.56E+06 ± 29.01%
unaligned reads (8.1%)	1.02E+06 ± 28.93%
**Grouping Quantity (Number of Identified RNAs)**	
< 10 rawcounts on average	2124
10 - 50 rawcounts on average	146
> 50 rawcounts on average	308
**Number of analyzed miRNAs**	
5 - 500 TPM	158
> 500 TPM	274

**Figure 6:**
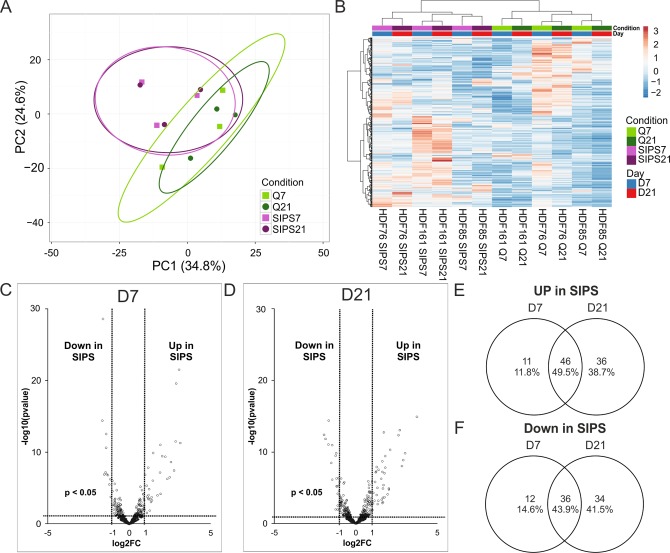
**Intracellular miRNA analysis by NGS identifies early and deep senescence specific miRNAs.** (**A**) Principal component analysis of SIPS versus Q HDF. Principal components were calculated using singular value decomposition (SVD) for imputation. Rows were scaled by applying unit variance scaling. Confidence level of 95% is indicated by ellipses assuming that a new observation from the same group will fall into it. Expression matrix of principal component 1 shows a variance of 34.8% and 24.6% in principal component 2. (**B**) Heatmap and hierarchical clustering of samples and miRNAs of SIPS versus Q human dermal fibroblasts. Clustering was done according to Euclidian distance and Ward linkage method. Samples in columns were clustered using correlation distance and Ward linkage method. (colors in matrix: red = highly transcribed = upregulated, blue = low transcribed = downregulated). (**C**) Volcano plot of differentially transcribed miRNAs in SIPS cells after seven (left D7) and (**D**) 21 days (right D21) post stress treatment. Log2FC are plotted on x-axis against their individual -log10 (p-value) on y-axis. Horizontal dotted lines indicate a separation between miRNA differences of a p-value higher or lower than 0.05. Vertical dotted lines separate transcribed miRNAs with log2FC > 1 or log2FC < 1. MiRNAs reaching a p-value < 0.05 are illustrated with white dots and miRNAs with a p-value > 0.05 are shown in black. (**E**) Venn diagram shows upregulated miRNAs of senescent cells on D7 and on D21. 46 miRNAs are commonly upregulated at both time points of senescence. (**F**) Venn diagram shows downregulated miRNAs of senescent cells on D7 and on D21. 36 miRNAs are commonly downregulated at both time points of senescence. (**A**-**D**) Analysis was performed using three different HDF cell strains (n = 3) each Q and SIPS from two different time points (D7 and D21). Differential expression analysis and statistics, calculated with Edge, was done with 432 miRNAs with normalized TPM signals > 5 in all conditions in at least 1 donor. (**A**-**B**) Each color and symbol represents another annotation defined by data input file. *Green*: Q; *Purple*: SIPS; *light colors and rectangular* D7; *dark colors and circle* D21.

Principal component analysis clearly separates senescent versus quiescent control cells independently from the time points (Fig. 6A), which was further confirmed by unsupervised hierarchical clustering (Fig. 6B).

Differentially transcribed miRNAs were identified (Supplementary List S5) and visualized by Volcano plot (Fig. 6C-D). Comparison of up- (Fig. 6E) and downregulated miRNAs (Fig. 6F) from early (D7) and deep senescent (D21) fibroblasts revealed senescence-associated miRNAs identified earlier, either in senescent fibroblast [35,36] or in the dermis of elderly [37], and thus point to a very robust miRNA signature of senescent fibroblasts. Surprisingly, a higher percentage of intracellular miRNAs (46% up and 36% down) are both regulated in early as well as in deep senescence (Fig. 6E-F), while in contrast to secreted ones per vesicle, only 2% and 5% are jointly increased or decreased (compare to Fig. 5C-D). That means that senescence-associated changes over time are more pronounced in secretory miRNAs (21 sEV-miRNAs of senescent cells change composition, Fig. S4B) as compared to intracellular ones (3 miRNAs differentially transcribed in senescent cells, Fig. S4C), so that these secreted miRNAs might be indicators of deep senescence.

We concluded that once senescence-signaling induces a specific intracellular miRNA pattern, it does not change significantly over time (Fig. S4C). Surprisingly however, the miRNA composition of secreted vesicles does change markedly with deepening of senescence (Fig. S4B), which might be attributable to the dynamic characteristics of the SASP [38–40].

### Correlation of intracellular and sEV-miRNAs identifies specifically secreted versus retained miRNAs in cellular senescence

Next, we addressed whether all miRNAs with high intracellular abundance are also highly abundant in sEVs and if this depends on senescence. Thus, after restrictive cut-off criteria (see Material and Methods for details), all miRNAs detected intracellularly and in vesicles (228 miRNAs) were ranked according to their abundance to build the intersection of the top 20 miRNAs each by Venn diagrams (Fig. S6A-B, Supplementary List S6). Thereby, we identified 26.5% matching miRNAs and it became clear that particular miRNAs must be selectively secreted or retained in the two conditions (Fig. S6C).

**Figure 7:**
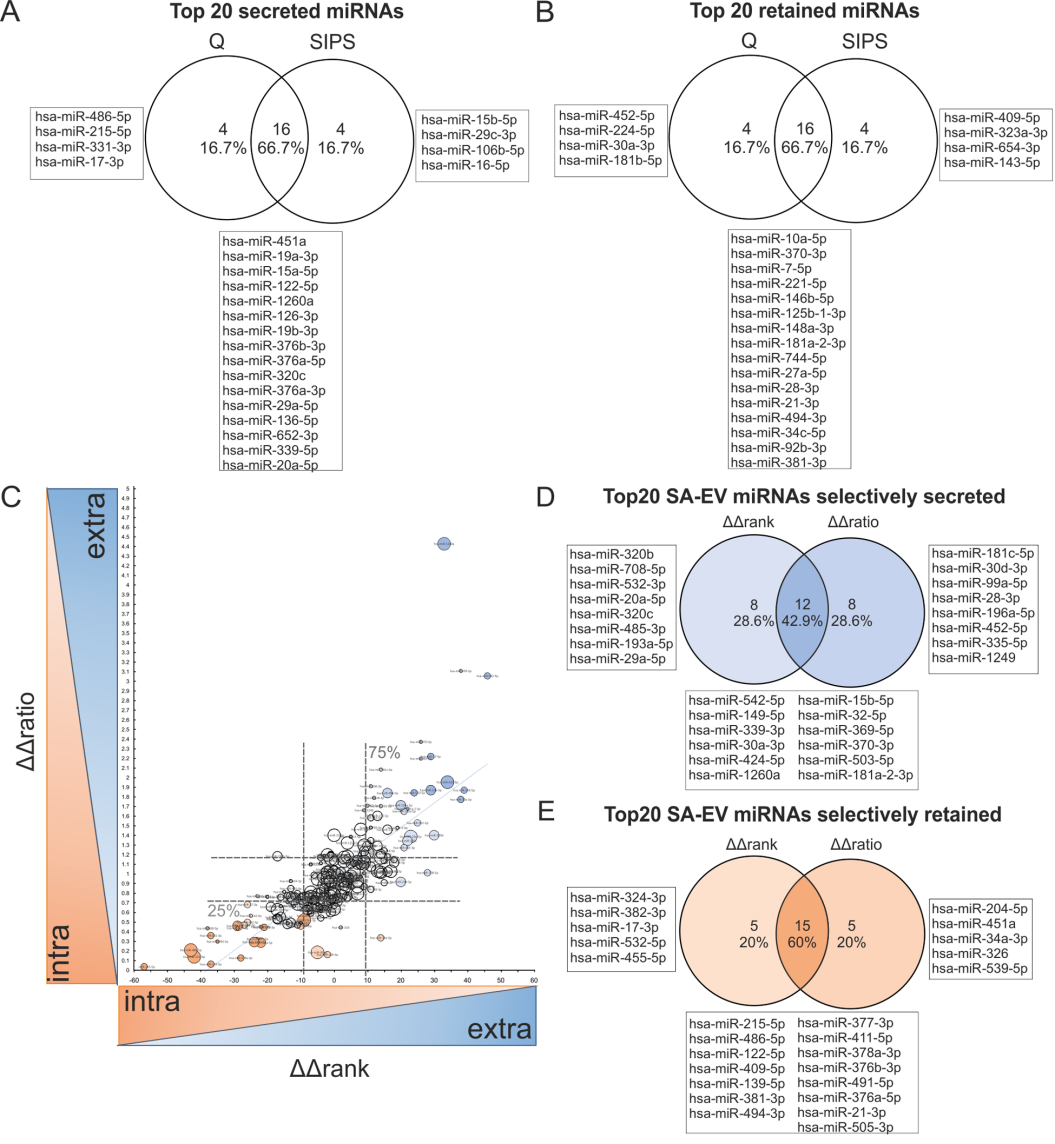
**Correlation of intracellular and sEV-miRNAs identifies specifically secreted versus retained miRNAs in cellular senescence.** (**A**) Venn diagram of top 20 secreted miRNAs (positive values) from HDF, calculated by Δrank = *rank*_intra_ – *rank*_extra_ from Q and SIPS separately. (**B**) Venn diagram of top 20 retained (negative values) miRNAs in HDF, calculated by Δrank = *rank*_intra_ – *rank*_extra_ from Q and SIPS separately. (**C**) Selectively senescence-associated secreted (high values) or retained (low values) miRNAs are identified. ΔΔrank and ΔΔratio were correlated and specifically secreted (high values of ΔΔrank and ΔΔratio) or retained (low values of ΔΔrank and ΔΔratio) senescence-associated miRNAs were identified. Spearman correlation R = 0.81 with a 95% confidence interval 0.76 to 0.85 P value (two-tailed) < 0.0001. Bubble size corresponds to quartiles calculated from transformed average Ct-values, whereby the larger the bubble size, the higher the expression value. Dotted lines represent the 25% and 75% percentiles, which define the specifically secreted and retained miRNAs in senescence. ΔΔrank: 25%: 8.0; Median: -0.5; 75%: 9.0; ΔΔratio: 25%: 0.7099; Median: 0.927; 75%: 1.186. (**D**) Venn diagram of the top 20 specifically secreted senescence-associated sEV-miRNAs. MiRNAs are identified by comparing the top 20 of ΔΔrank and ΔΔratio method. (**E**) Venn diagram of top 20 specifically retained senescence-associated miRNAs. MiRNAs are identified by comparing the top 20 of ΔΔrank and ΔΔratio method. (**F**) (**A**-**E**) Correlation was performed with 228 miRNAs identified intracellularly (small RNA-NGS) as well as in sEVs (qPCR panels) in samples derived from three different HDF cell strains (n = 3) each Q and SIPS from two different time points (D7 and D21).

In order to identify those miRNAs that are differentially packaged or retained in cells, we calculated the differences of ranks of the intracellular and the sEV contained miRNAs (Δrank = *rank*_intra_ – *rank*_extra_) for quiescent and senescent cells separately (Supplementary List S6, S7, Fig. 7A-B). Then the ΔΔrank(Δ*rank*_SIPS_ – Δ*rank*_Q_) were calculated, as a measure to indicate, if a miRNA would change its rank in dependence of the cell condition. That means, the higher the ΔΔrank-value is, the higher is also the selective secretion, and vice versa, the lower the value, the higher the specific retention within the cell (Supplementary List S6, Fig. S6D). Thereby, we identified specifically secreted senescence-associated sEV-miRNAs, such as miR-15b-5p (Fig. 7A), while miR-30a-3p was found to be retained during quiescence (Fig. 7B).

In order to confirm the ΔΔrank correlation by a different method to assess specific secretion, we additionally used the quantitative content of our intracellular and vesicular miRNA data. Therefore, we calculated ratios between vesicular and intracellular miRNA levels, and used these values to calculate ratios from quiescent (Δ*ratio*_Q_) and senescent cells (Δ*ratio*_SIPS_). Therefore, we transformed Ct-values to arbitrary units (AU), by defining a Ct-value of 40 to 10 AU. We then calculated ΔΔ*ratios*(Δ*ratio*_SIPS_/Δ*ratio*_Q_) and normalized obtained values to the global means, resulting again in a list of specifically secreted or retained miRNAs (Supplementary List S6, S7 Fig. S6E).

To compare these two methods (‘rank vs. ratio’), the results were plotted in an xy-diagram revealing ~80% of correlation as determined by Spearman correlation (Fig. 7C) and comparison of the top 20 selectively secreted and retained miRNAs each confirmed a similar set of the top 20 selectively secreted (Fig. 7D) or retained miRNAs after entry into cellular senescence (Fig. 7E), while some miRNAs were only detected with one of the two methods. Thereby, miR-15b-5p was again identified to be selectively secreted from senescent cells. Finally, by defining a cut-off of the 25% and 75% percentiles from both approaches, we identified ~24% of all analyzed miRNAs to be selectively secreted (blue) or retained (orange) in response to senescence, while the remaining ones seem to be evenly distributed between cells and sEVs (white). Interestingly, although miR-21-5p is the top abundant miRNA intracellularly as well as in vesicles, independent of the conditions, and therefore equally distributed between inside and outside (Supplementary List S6, S7 and Fig. S6A-B), its 3p-isoform was catalogued as a selectively retained miRNA during senescence.

To sum up, the ‘ratio-‘ and ‘rank-’ approaches allow the correlation of vesicular versus intracellular miRNA abundance, independently from each other and identified a set of specific senescence-associated miRNAs selected for secretion (blue) or retention (orange) in response to senescence.

## DISCUSSION

Accumulation of senescent cells is considered to drive several age-associated diseases. One of the characteristic of senescent cells that is considered to contribute to this phenomenon, is the cumulative secretion of several proteins involved in inflammation, growth promoting signaling and extracellular matrix remodeling, which is generally summarized under the term SASP [12]. With increasing numbers of reports on secretory miRNAs describing their almost ‘hormonal’ action on recipient cells [15] and their potential as biomarkers or therapeutic targets for age-associated diseases [20], the question arises whether secreted miRNAs, especially those enclosed in EVs, might also be part of the SASP.

Indeed, we found a 4-fold higher secretion of sEVs from senescent as compared to quiescent human dermal fibroblasts with a concomitant increase of > 80% of all miRNAs per cell, whereby the stress-responsive miR-200c-3p [41,42] was found to be among the top differentially secreted miRNAs at an early time point of senescence. An increase of EVs in replicative senescence, as well as in irradiation-induced senescent prostate cancer cells [43] has already been observed. Even in human age-associated diseases, as in human atherosclerotic aortas [44], or in cerebrospinal fluid of Alzheimer’s disease patients [45] where senescent cells have been found to accumulate *in vivo,* such an increases of EVs was evident. However, decreasing amounts of EVs with age have also been observed in the plasma of matched individuals [46]. It will thus be exciting to see, where and if EVs are differentially distributed between lesional sites of age-associated diseases versus the aged systemic environment.

As a consequence of elevated sEV and miRNA secretion per senescent cell, we performed pathway analysis of the most highly secreted miRNAs enclosed in the sEVs. Surprisingly, they were predicted to collectively silence five well known pro-apoptotic factors [11,29–33] at the crossroad of longevity, cancer and signalling pathways. Indeed sEVs from senescent fibroblasts reduced the amount of apoptotic cells in acutely stressed recipient fibroblasts. Even though the single factors of the sEVs are yet not identified, we postulate that the secretion of anti-apoptotic sEVs into the microenvironment of senescent cells might counteract the apoptotic removal of damaged neighbouring cells, thereby potentially contributing to a pro-tumorigenic microenvironment as known to be conferred by senescent cells and their EVs [47,48]. EVs per se have already been suggested to exert anti-apoptotic functions on the surrounding tissue and cells [49], however, this is to our knowledge the first report that experimentally proves that the SASP, and specifically the EV-SASP exerts anti-apoptotic activity. This is in line with a bioinformatic driven study of the protein factors comprising the SASP that postulates a potential anti-apoptotic activity of SASP proteins [50]. However, it is still to be determined, which miRNAs or if the entire cocktail of secreted miRNAs are indeed conferring this activity.

In addition, we identified differences in miRNA composition per single vesicle from senescent versus control cells. In accordance to being upregulated intracellularly in senescent fibroblasts [51,52] we found miR-23a-5p and miR-137 to be more abundant per vesicle. Among the less abundantly present miRNAs in senescent sEV we found miR-17-3p and miR-199b-5p, both were already published to be downregulated intracellularly in skin of elderly [53] and in senescence of mesenchymal stem cells [54].

Similarly, intracellular miRNA transcription of senescent versus quiescent fibroblasts, revealed similar miRNAs that have been previously reported in fibroblasts [55–58], as well as miRNAs differentially found in the dermis of elderly [37], where estimates suggest 60% of fibroblasts to be senescent [59]. In addition, several miRNAs were identified so far not yet described in fibroblast senescence, such as miR-1197 and miR-450-2-3p.

With intracellular and extracellular miRNA quantitative data in hand, we next tested, if (i) specific miRNAs are selectively packaged into sEVs or retained within fibroblasts as it has been reported for other cell types [60] and (ii) if this is dependent on senescence, which has so far never been tested. Therefore, we ranked the abundance of miRNAs in- and outside of the cells and compared the resulting ranks. Most of the miRNAs, such as miR-21-5p are similar in rank, suggesting that most of the sEV cargo is mirroring the cytoplasmic content of the respective cell, while some miRNAs are indeed overrepresented intracellularly or in the sEVs. However, these specifically retained or secreted miRNAs were only partially overlapping when comparing the senescent and quiescent cells, suggesting that upon induction of senescence, also specific packaging or retaining does change, which was indeed the case for ~24% of all analyzed miRNAs.

It is still a matter of debate, if packaging of selected miRNAs into EVs is an active process for conveying messages or a passive form of garbage disposal [discussed in 42] e.g for the secretion of damaged RNA fragments [62] or for the release of tumor-suppressive miRNAs to maintain tumor progression [63]. However, our findings, together with the few reports that show specific retaining or packaging in response to external stimuli [64] and changes in EV-miRNA composition of PBMCs [65] would suggest controlled and active packaging, in line with several reports showing an active mechanism of miRNA packaging into exosomes [60,66]. Finally, as miRNAs in EVs have been widely shown to alter recipient cell behavior [17,18] a mere garbage disposal seems unlikely, while it could be envisaged that ‘garbage’ gotten rid of by one cell might be an alarm - or any other type of signal for recipient cells.

Which miRNAs are now selectively secreted by senescent cells and what effect on the microenvironment might such specifically packaged miRNAs have?

One of these is miR-15b-5p, which we found to be selectively secreted and downregulated in senescence as it was reported before in senescent fibroblasts as well as in photoaged skin biopsies [67]. The fact that it is preferentially packaged and secreted in senescence might be an additional mechanism to keep miR-15b-5p levels low in senescence cells. Interestingly, it is also low abundant in the dermis of elderly, while it appears highly enriched in the epidermis [67]. Thus, it is tempting to speculate that EV mediated cross-talk between fibroblasts and keratinocytes contributes to low dermal levels versus high epidermal levels. Functionally, low intracellular miR-15-5p levels might be involved in de-repressing SIRT4, which has a regulatory role in stress-induced senescence-associated mitochondrial dysfunction [67] and in driving a NF-кB mediated induction of the SASP [68]. On the other hand, it might exert pro-proliferative activity on recipient keratinocytes as it does on several epithelial cell types [69], a function that in situation of transient appearance of senescent cells during wound healing might be favorable [70], while in situations of chronic accumulation of senescent cells, as in the skin of elderly, it might be detrimental.

Interestingly, several miRNAs mainly selectively retained in senescence including miR-122-5p [71], miR-21-3p [72] and miR-17-3p [73] are implicated with keratinocyte differentiation and/or proliferation, suggesting that senescent fibroblasts might impact on epidermal differentiation and function.

Taken together, we conclude that miRNAs are specifically secreted depending on cellular conditions and/or external stimuli. The specific molecular mechanism of selective release and retention of senescence-associated sEV-miRNAs and the EV-SASP cross talk between different cell types and its consequences in the context of aging and age-associated diseases, however, remains to be elucidated. Still, the here presented detailed catalogue based on human dermal fibroblast strains derived from three different donors builds the basis for such studies. Finally, we introduce sEVs and sEV-miRNAs as novel, *bona fide* members of the SASP to be crucially involved to maintain the anti-apoptotic activity of senescent cells and suggest to use the term ‘EV-SASP’.

## METHODS

Detailed experimental procedures are provided in the supplementary information.

### Cell culture

Human dermal fibroblasts (HDF) from adult skin of three healthy donors and human foreskin fibroblasts of one healthy donor were provided by Evercyte GmbH. Cells were grown in DMEM/Ham’s F-12 (1:1 mixture) (BIOCHROME, Germany) supplemented with 10% fetal calf serum (FCS) and 4 mM L-Glutamine (Sigma Aldrich GmbH St Louis, MO, USA) at 95% air humidity, 7% CO_2_ and 37°C.

### Stress-induced premature senescence (SIPS)

For induction of SIPS, the Hayflick limit of each of the here used donors was assessed and cells in the middle of their replicate life span were used. Two donors (HDF161 and HDF85) reached the end of their replicative lifespan very early. HDF161 at a PD of ~ 37, HDF85 at a PD ~ 28, while HDF76 entered replicative senescence at a PD ~ 53. Therefore, HDF161 and HDF85 in PD ~ 12 – 15 and HDF76 in PD ~ 24 – 26 were seeded with 3500 cells/cm^2^ one day (d) prior stress treatment using 9 (4 d stress – 2 d recovery – 5 d stress) consecutive doses of 100 µM H_2_O_2_ for one hour per day followed by a media change. Non-stressed control cells reached quiescence (Q) by contact inhibition.

SIPS was confirmed with bromodeoxyuridine (BrdU) incorporation, senescence-associated (SA)-ß-Gal staining, CDKN1A (p21) expression and Annexin-V-PI staining after 7 (D7) and 21 days (D21) post stress treatment. See supplementary Information for detailed experimental procedures.

### Isolation of small extracellular vesicles (sEVs)

Small EV Isolation was performed according to standards recommended from the international society for extracellular vesicles (ISEV) [74]. DMEM/Ham’s + FCS was depleted of EVs by ultracentrifugation at 100,000 x g overnight and filtrated using 0.22 µm filter cups (MILLIPORE, Germany). Conditioned media (after 48 hours secretion) was centrifuged for 15 min at 500 x g (Eppendorf, 5804R) to remove cellular debris at 14,000 x g (Beckmann, Coulter, Brea, CA, USA, Avanti JXN-26) for 15 min, large EVs were excluded by filtration using 0.22 µm filter cups. On average 92 ml supernatant from SIPS and 75 ml supernatant from Q cells were filled into Quick-Seal, Polyallomer, 39 ml, 25x89 mm tubes (BECKMANN, Brea, CA, USA). SEVs were enriched using a 70Ti Rotor Beckman coulter at 100,000 x g for 90 min (BECKMANN, Brea, CA, USA) and pellets in different tubes but from the same samples were pooled. Dependent on the subsequent analysis, the pellet was either resuspended in QIAzol reagent (Qiagen) or in filtered 1 x PBS. For TEM freezing and thawing was avoided. SEVs were isolated on D7 and D21.

### Biological assay – exposure to sEVs and stress treatment

To test the biological effect of the EV-SASP, we selected early passage human dermal and foreskin fibroblasts as recipient cells. After 48 hours secretion into EV depleted media, the sEVs from SIPS and Q donor cells were freshly harvested from all three different fibroblast cell strains between two to four weeks of cellular senescence. The experiment was performed as followed (Fig. 4B):

D -1 EV depleted media was added to donor cells of SIPS and Q fibroblasts for 48 hours before sEV harvesting.

D 0 recipient dermal or foreskin fibroblasts were seeded into 6 well plates with 70,000 cells/well.

D 1 sEVs of SIPS and Q donor cells were harvested and recipient fibroblasts were pre-treated with sEVs in a ratio of 1:1 (meaning same amount of secreting cells to receiving cells).

D 2 sEVs were removed and recipient cells were treated with 200 µM H_2_O_2_ for 2 hours. Afterwards fresh sEVs were added again.

D 3 sEVs were removed and recipient cells were treated with 400 µM H_2_O_2_ for 2 hours followed by a recovery of 3 hours. Finally, the cells were stained for Annexin-V and with PI and were measured by flow cytometry (Gallios Beckman coulter, Brea, CA, USA). As a positive control, fibroblasts were treated with 300 nM Staurosporin for 24 hours. Total amount of apoptotic cells correspond to: Annexin positive + double positive (Annexin-V-PI) + PI positive cells and were quantified using Kaluza software (Beckman Coulter, Brea, CA, USA, Version 1.2).

### RNA Isolation

Cell pellets and sEVs were lysed in QIAzol Reagent (QIAGEN) and RNA was automatically extracted by miRNeasy Mini kit (QIAGEN) based on QIAcube technology. To monitor isolation efficiency of sEV-RNA, a spike-in mix containing UniSp2, UniSp4, UniSp5 (EXIQON, Denmark,) was added before RNA isolation. As total sEV-RNA amounts were too low for quantification by Bioanalyzer (Agilent) or with comparable, more sensitive techniques such as Ribogreen assay, we normalized the data (i) to total viable cell number and (ii) to the global means of each, which is an accepted method, not only in EV-research [34,74]. No significant differences in the global means of different samples were observed (Fig. S4A).

Intracellular total RNA concentration and quality was controlled using Nanodrop spectrometer (ND-1000) and 2100 Bioanalyzer (Agilent) using the RNA-6000 Nano Kit. Average RNA concentration as determined by Nanodrop and Bioanalyzer revealed average concentrations as followed: For Q = 955 ng/µl and for SIPS = 234 ng/µl purified in a volume of 20 µl NFW. RIN of intracellular RNAs was determined by 2100 Bioanalyzer, revealing for Q = 7.3 and for SIPS = 7.5. For cDNA library preparation 1 µg of total RNA was used.

### cDNA synthesis

Equal volumes of sEV-RNA were used for cDNA synthesis using Universal cDNA Synthesis Kit II (EXIQON, Denmark). UniSp6 and cel-miR-39 (EXIQON, Denmark) were used to control for enzyme activity. cDNA was synthesized by 42°C for 60 min followed by heat inactivation for 5 min at 95°C.

For mRNA quantification, cDNA was synthesized from 500 ng of total RNA with the High-Capacity cDNA Reverse Transcription Kit including RNAse inhibitor, (APPLIED BIOSYSTEMS, USA) for 10 min at 25°C - 120 min 37°C - 5 min 85°C.

### Quantitative Real Time PCR (qPCR)

MiRNA qPCR analyses were performed using ExiLENT SYBR® Green master mix and LNA-enhanced miRNA primer (EXIQON, Denmark) on a LC 480 Real Time PCR system (ROCHE, Germany). Activation: Cycles 1, Analysis Mode: None, 95°C, 10min, Ramp 4.4°C/s. Cycles: Cycles 45, Analysis Mode: Quantification 95°C, 10s, Ramp 4.4°C/s, 60°C, 60s, Acquisition Mode: Single, Ramp 1.6°C/s. Melting Curve: Cycles 1, Analysis Mode: Melting Curves, 95°C, 10s, Ramp 4.4°C/s; 55°C, 60s, Ramp 2.2°C/s; 99°C, Acquisition Mode: Continuous, Ramp 0.11°C/s, Acquisition per °C: 5. Cooling: Cycles 1, Analysis Mode: None. The second derivative method was used to calculate the cycle of quantification values (Ct-values).

The microRNA, Ready-to-Use PCR, Human panel I+II, V3.R, EXIQON, Denmark, were used for a preliminary screening. Based on that, a customized qPCR panel was designed comprising 375 miRNAs and internal and negative controls.

QPCR for mRNA was performed with 5x HOT FIREPol® EvaGreen® qPCR Mix Plus with ROX (MEDIBENA, Austria) using a Rotor-GeneQcycler. Determination of CDKN1A and GAPDH was quantified using Standard curves for determination of copy numbers in duplicates. Average expression values from quadruplicates were normalized to GAPDH as a reference gene and fold changes were calculated.

Negative controls tested as NFW only, and no template control derived from cDNA synthesis, were below detection limit of qPCR (> 40). Primer used for qPCR is presented in Table 4.

**Table 4:** Primer used for qPCR.

**Gene name**	**Sense primer**	**Anti-sense primer**
GAPDH	CGACCACTTTGTCAAGCTCA	TGTGAGGAGGGGAGATTCAG
CDKN1A (p21)	GGCGGCAGACCAGCATGACAGATT	GCAGGGGGCGGCCAGGGTAT

All analyses were performed in biological triplicates in two conditions (Q and SIPS) and two time points (D7 and D21). In total, 12 qPCR panels were set up on three consecutive days. MiRNA analysis was performed according to the ddCT method.

### qPCR panel, analysis of sEVs-miRNAs

Spike-ins were detected in all 384-well plates to monitor purification efficiency of RNA Isolation (UniSp2, UniSp4, UniSp5), the presence of enzyme inhibitors during cDNA synthesis (Unisp6 and cel-miR-39–3p) and equal processing of RT-qPCR amplification (interplate calibrator IPC - UniSp3). NFW was used to determine background levels of each miRNA. Constant expression of all spike-ins was evaluated with a range calculated by the difference of the highest and lowest value of all samples/plates. ΔCt_r_ values below 1 define the experiment to be robust and thus allow the exclusion of inter-assay variations (for details, see manual from EXIQON, QC PCR panel #203887-203892, September 2014).

### Illumina small RNA library preparation

Intracellular small RNA cDNA library for Illumina Sequencing was synthesized according to the manual provided by NEBNext® Small RNA Library Prep Set for Illumina® (Multiplex Compatible) (NEB, E7330S). From initially 1 µg of total RNA, small RNA fragments from approximately 18 – 36 nucleotides were gel purified on a 10% TBE Gel (Invitrogen/ Thermo Scientific, EC62752), quantified by 2100 Bioanalyzer (Agilent, Santa Clara, CA, USA) and equimolar amounts were pooled and sent to Exiqon (Denmark) for Illumina RNA-Seq.

RNA isolation and cDNA library preparation were quality controlled prior to NGS (Fig. S5A-C). After adapter trimming and mapping, on average 17.6 million reads per sample were obtained (Fig. S5G-H). The entire dataset was evaluated (Table 3 and Fig. S5D-F), normalized to the number of total reads and 432 miRNAs that reached at least five tags per million (TPM) in one donor were included into the analysis.

### Illumina, miRNA next generation sequencing (NGS)

The cDNA library pool was used to generate the clusters on the surface of a flowcell and NGS was performed using NextSeq 500 (EXIQON, Denmark). The collected reads were quality controlled, aligned and identified miRNAs were annotated to miRBase20 by Exiqon.

### Differential expression analysis of NGS data

Differential expression analysis was done using the R (version 3.2.2)/Bioconductor software package DESeq [75]. Low expressed miRNAs were first excluded from the analysis (TPM < 5 for all the samples). Then, the raw read counts were normalized using the DESeq normalization and a model based on negative binomial distribution and local regression was fitted for each miRNA. In the model. ‘fibroblast cell strains (n = 3)’, were defined as a block ‘effect’ and ‘day’ and ‘condition’ as factor of 2 levels. The Benjamini and Hochberg (BH) procedure [76] was applied to adjust the raw p-values into false discovery rate (FDR). A FDR < 0.05 was chosen as the cut-off value.

### Differential expression analysis of qPCR panels of sEV-miRNAS

Ct-values were either normalized to total number of cells used for secretion or by the mean-centering restricted (MCR) normalization [34,77], also known as the global mean normalization. Thereby, the mean Ct-value across all detected miRNAs of a single sample was subtracted from each individual miRNA. Differences in global means are presented in Fig. S4A.

Both datasets were subjected to differential expression analysis with the R (version 3.2.2)/Bioconductor software package Limma [78]. A linear model was applied for each miRNA and moderated t-tests were computed. In the model, ‘fibroblast cell strains (n = 3)’, were defined as a ‘block effect’ and ‘day’ and ‘condition’ as factor of 2 levels. The raw p-values were corrected using BH method to control FDR.

### Statistical analysis

#### Routine statistics

Were either calculated with Excel or Graph Pad Prism, and respective tests are indicated below figures in result sections. Averages +/- standard error (SEM) or deviation (STDEV) were derived from at least 3 independent experiments. Two tailed tests were performed using an error probability of 0.05.

Data were tested for Gaussian distribution if possible. If normally distributed, two groups were compared using unpaired or paired student T-test using the raw values. One sample students T-test was used to compare ratios to a hypothetical value of 1, respectively. In order to analyze the impact of two independent factors (for example ‘treatment’ and ‘day’) a two-way repeated measures (RM) ANOVA was performed followed by Bonferroni post test if asked.

#### Descriptive statistics

ClustVis a web tool for the preparation of principal component 2D-biplots and heatmap analysis based on multivariate datasets using different R packages was used [79]. For all exploratory analyses, normalized Ct-values and TPM values were used. Principal component analysis (PCA) of 371 extracellular miRNAs (out of 375) was calculated by iteration of missing values with Nipals PCA and unit variance scaling was applied to rows. Heatmap preparation and unsupervised hierarchical clustering of secreted miRNAs was performed by applying correlation distance and Ward linkage. Samples in columns are clustered using Euclidean distance and Ward linkage method.

PCA for intracellular miRNAs was done for 432 miRNAs with TPM > 5 in at least one donor. We used Singular Value Decomposition (SVD) for imputation and unit variance scaling was applied on TPM values. Expression matrix and unsupervised hierarchical clustering of 432 intracellularly transcribed miRNAs was done by applying unit variance scaling and rows were clustered using Euclidean distance and Ward linkage. Columns are clustered using correlation distance and Ward linkage.

### Correlation of intracellular and vesicular miRNAs

Only miRNAs, included in the customized qPCR panels for determination of vesicular miRNA abundance (375) and corresponding intracellular miRNA expression obtained by NGS were selected. Prior correlation of intracellular and vesciular miRNAs restrictive cut-off criteria were applied.

Quartiles from Ct-values and TPM values were calculated and miRNAs being low expressed (quartile 1 corresponds to the lowest 25% of data) in NGS and qPCR were excluded from analysis (330 miRNAs). Then miRNAs giving no signal in NGS experiment (TPM = 0) were excluded (291 miRNAs), and finally all miRNAs not present in all three donors and conditions were excluded. Therefore, correlation was done on 228 miRNAs.

In order to reduce sequence specific bias obtained with NGS and qPCR, we calculated the differences in retaining versus specific secretion by 2 different approaches; (i) by ranking the miRNAs and calculating the change in rank within the NGS and the qQPCR datasets;(ii) by calculating the abundances via ratios. The overlap of both methods is presented as result and considered to be a strict way of analysis which rather takes the risk to miss some miRNAs than to provide false positives.

In detail: ranks from averages were calculated from SIPS and Q separately. Rank order was done according to intracellular TPM values to identify most abundant miRNAs transcribed intracellularly, or according to vesicular Ct-values, to discover most abundantly present miRNAs in sEVs. By calculating Δrank (rank_intra_ – rank_extra_) from Q and SIPS separately, retained (negative value of Δrank) and secreted miRNAs (positive value of Δrank) were identified. By further calculating ΔΔrank(Δrank_SIPS_ – Δrank_Q_) and the 25% and 75% percentiles, selectively higher secreted (high value of ΔΔrank) or retained (low value of ΔΔrank) miRNAs in SIPS were discovered.

Next, we analyzed the same dataset with a different method to review our data, using the ‘ratio-approach’. For a better visualization, Ct-values were transformed to arbitrary units, defining a Ct-value of 40 to ‘10’ arbitrary units – assuming around 10 miRNA copies. Δratios were calculated from values intra_SIPS_/extra_SIPS_ and intra_Q_/extra_Q_ separately. Then ΔΔratios from Δratio_SIPS_/Δratio_Q_ were calculated and normalized to the global mean of those ratios. Again, the 25% and 75% percentiles were calculated, and selectively higher secreted (high value of ΔΔratio) or retained (low value of ΔΔratio) miRNAs in SIPS were discovered. For Fig. S6D miRNAs were sorted according to ΔΔrank values from smallest to largest values and they were plotted on y-axis, ΔΔratio values were then plotted in another diagram (Fig. S6E) in the same order as it was sorted before.

### Pathway analysis of secretory miRNAs

MiRWalk ‘microRNA- gene target’ tool [80] was used to find all validated targets for each of the 20 most highly secreted miRNAs. To evaluate the putative network on pathway level, enrichment analysis of pathway-based sets of the common regulated genes (targets) was performed using ConsensusPathDB [81], with the overrepresentation analysis tool. As input, HGNC symbol identifiers of our dataset were used and search was done against pathways with a minimal overlap of a p-value cutoff of 0.0001. Cytoscape [82] and the BisoGenet plug-in [83] was then used to generate a potential miRNA-regulated network using the list of validated targets and the modules obtained in the previous step. Crosstalk maps were created, linking curated pathways to metapathways [84–86] where several pathways modules share a common set of genes.

### Accession Number

miRNA NGS data from differentially transcribed miRNAs in stress-induced premature senescence (SIPS) have been deposited to the GEO repository under the accession number GSE95354 https://www.ncbi.nlm.nih.gov/geo/query/acc.cgi?token=ytojsgmknpqbtix&acc=GSE95354

## Supplementary Material

Supplementary Figures, Methods, References

Supplementary Data List
