# Supplementary material for: Small extracellular vesicles and their miRNA cargo are anti-apoptotic members of the senescence-associated secretory phenotype
Source: Aging (Albany NY). 2018 May 19;10(5):1103–32. doi: 10.18632/aging.101452 (PMC5990398; doi:10.18632/aging.101452)
Supplement: Supplementary Figures, Methods, References [file aging-10-101452-s001.pdf]

SUPPLEMENTARY MATERIAL

Supplementary Figures

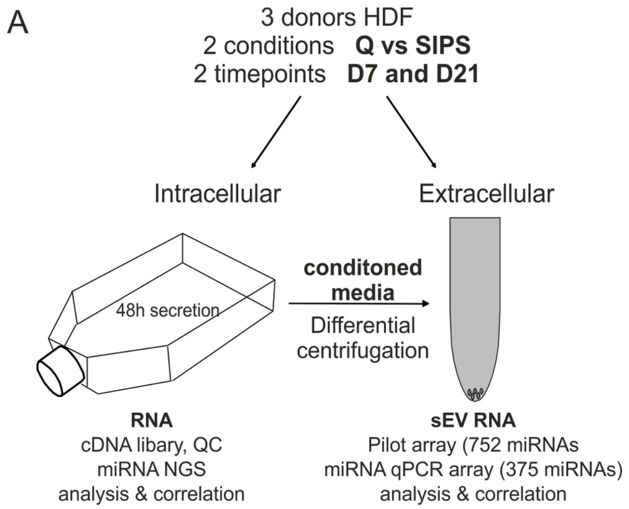

Figure S1. Scheme of experimental workflow. (**A**) SIPS was triggered in three donors of primary human dermal fibroblasts' (HDF) by chronic low doses of H_2O_2. Seven days (D7) and 21 days (D21) after the last H_2O_2 pulse, intracellular RNA was harvested and cDNA library for small RNA NGS was synthesized. Correspondingly, sncRNA from small extracellular vesicles (sEVs) was isolated from conditioned supernatants by differential centrifugation. SEV-RNA was prepared for qPCR panels to identify senescence-associated sEV-miRNAs.

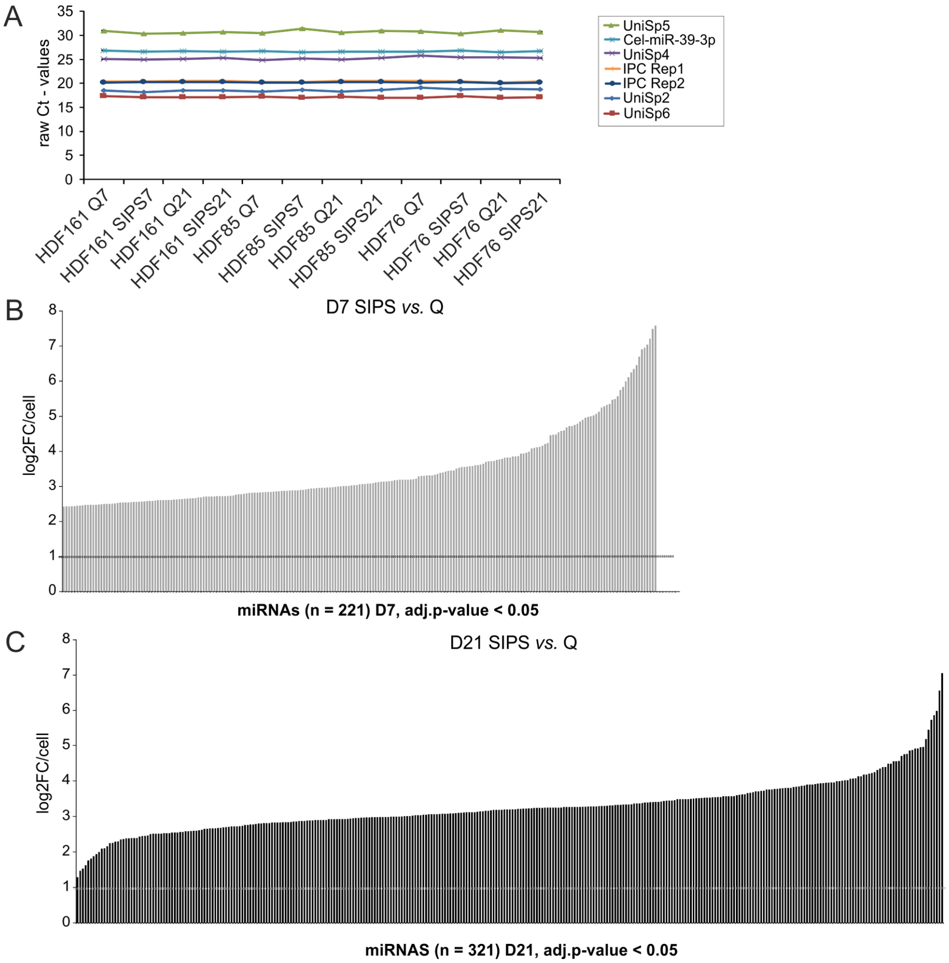

Figure S2. Data quality control and analysis of miRNAs enclosed in small extracellular vesicles. (**A**) Quality control using synthetic RNA-spike-in confirms technical coverage (ΔCt_r values below 1) of screening comprising 12 samples of vesicular RNA from three different donors and two different time points (D7 and D21). Each qPCR plate contained primer for synthetic spike in RNAs that were added during RNA isolation (Unisp2, Unisp4, Unisp5) and cDNA synthesis (Unisp6, cel-miR-39). Additionally, each panel included two interplate calibrator (IPC) and an empty negative control. (**B**) Bar chart of significantly higher secreted miRNAs of SIPS HDF on D7 after the treatment. Log2FC values from three biological triplicates were calculated and plotted on y-axis. Bars plotted on y-axis show all miRNAs reaching an adjusted p-value < 0.05 after applying the BH method for FDR. On D7, 221 EV-miRNAs passed the adjusted p-value. Dotted lines represent log2FC = 1. (**C**) Bar chart of significantly higher secreted sEV-miRNAs of SIPS HDF on D21 after the treatment. Log2FC values from three biological triplicates were calculated and plotted on y-axis. Bars plotted on y-axis show all miRNAs reaching an adjusted p-value < 0.05 after applying the Benjamini Hochberg method for FDR. On D21, 321 EV-miRNAs passed the adjusted p-value. Dotted lines represent log2FC = 1.

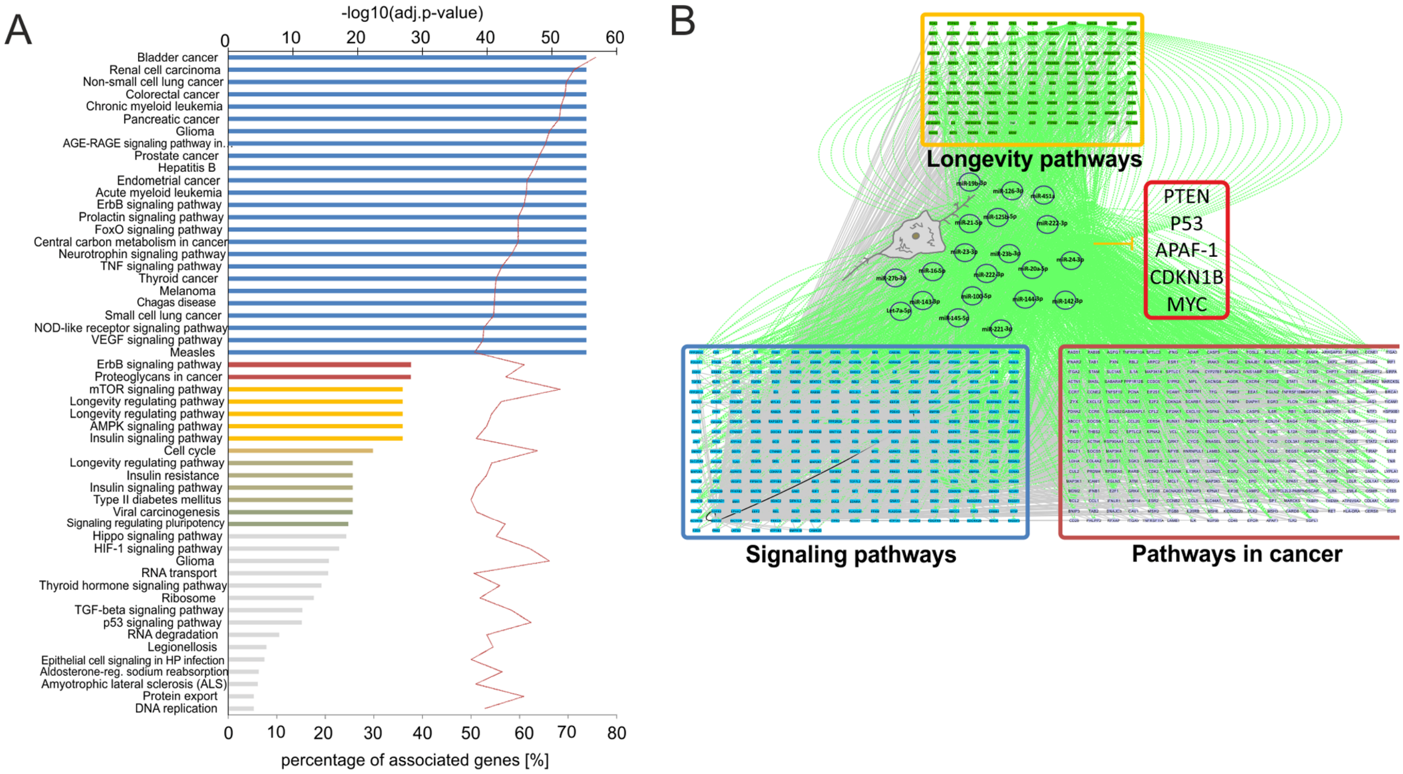

Figure S3. Pathway analysis of the EV-SASP. (**A**) Bachart shows 54 network modules with more than 50% of all associated genes targeted by the 20 most abundantly secreted miRNAs with an adjusted p-value < 0.0001. GO terms are plotted against their -log10(adj.p-value). Red line indicates the number of associated target genes identified within all interactions (5,437 validated targets were identified). Color of barcharts (blue, red, yellow, olive, avocado green, gold) correspond to one GO group that contain more GO-Terms. Grey bars correspond to different GO groups that contain only one GO term. Abbreviation HP: Helicobacter pylori infection. (**B**) Top20 secreted miRNAs regulate a dynamic crosstalk of three prominent metapathways and five common transcription factors (PTEN, P53, APAF-1, CDKN1B and MYC). Several gene modules were detected to participate repeatedly in several pathways, indicating a crosstalk of pleiotropic genes and various gene modules involved in series of cellular activities. Based on that finding, large metapathways identified a complex network that pinpoints towards an interplay between signaling, longevity and cancer pathways, which are supposed to be orchestrated by the secreted miRNAs and their target genes suggesting a potential anti-apoptotic activity of the EV-SASP on target cells. Longevity pathways, signaling pathways and pathways in cancer are shown. Green edges represent miRNA regulation over their targets across different pathways. Grey edges represent protein-protein interactions and transcriptional regulation. Graphic illustrates the top 20 highly secreted miRNAs commonly targeting five transcription factors.

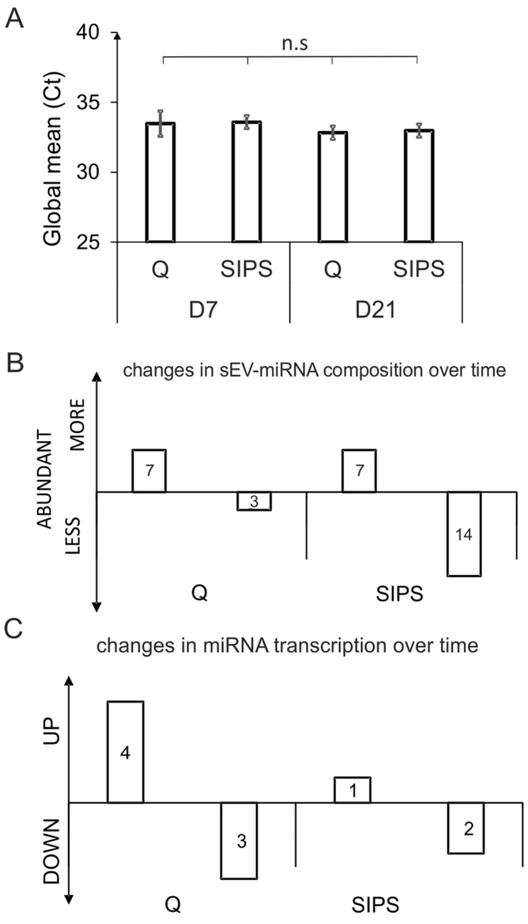

Figure S4. Calculation of Global means and changes in miRNA abundances over time in vesicles and intracellularly. (**A**) Global mean used for normalization. Averages of three different HDF strains +/- STDEV is shown. 2-way ANOVA was used to test for condition ($p = 0.73$) and day ($p = 0.11$); (n.s) $p > 0.05$. (**B**) 21 sEV-miRNAs of SIPS cells change their composition over time. Global mean-normalized Ct-values from biological triplicates were averaged and log2FC relative to day 7 recovery were calculated (p-value < 0.05). (**C**) 3 miRNAs are differentially transcribed in SIPS cells over time. Intracellular miRNA transcription relative to day 7 was calculated from NGS data. miRNAs with an adj. p-value < 0.05 were taken into account.

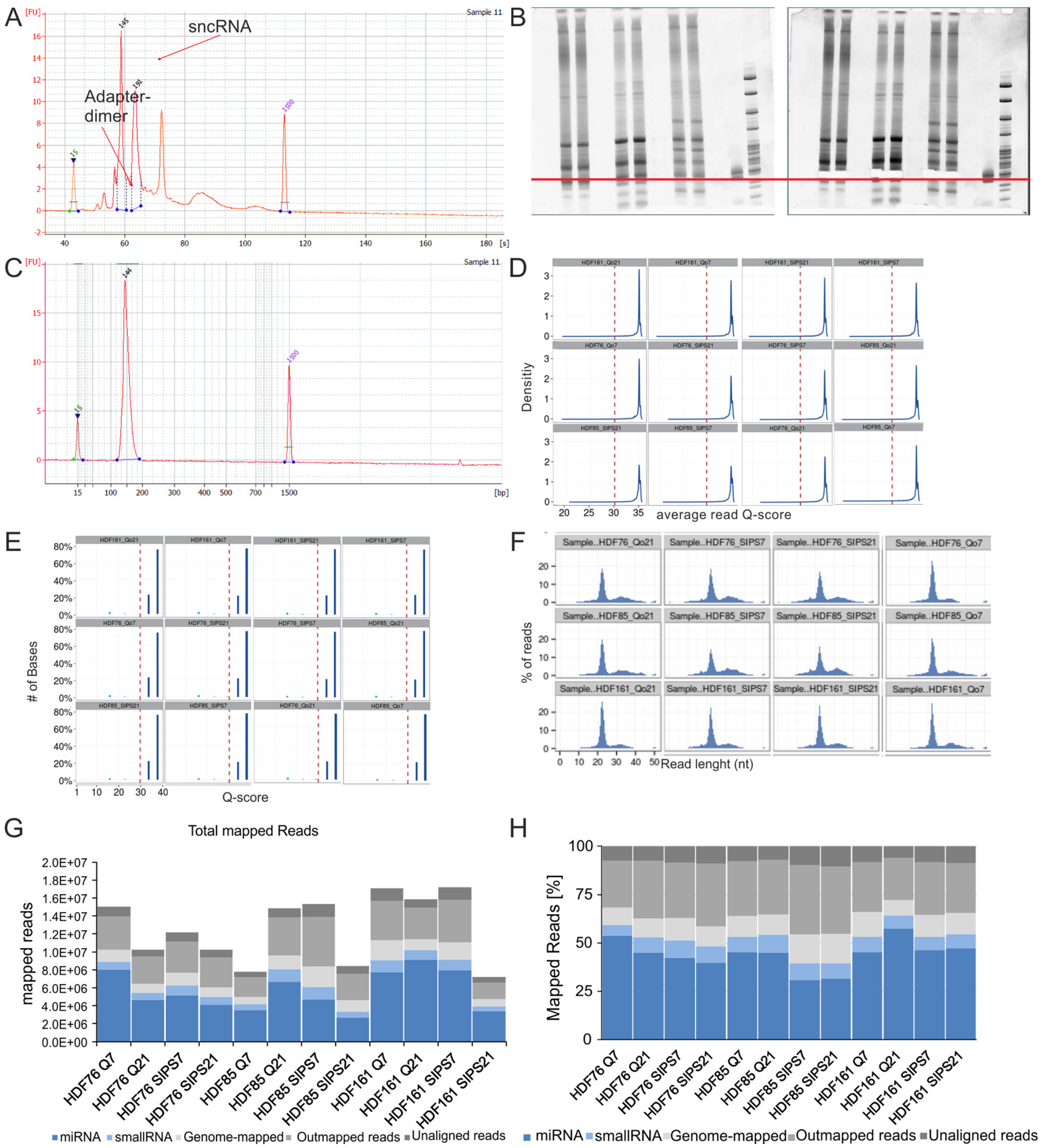

Figure S5. Data quality control of cDNA library preparation and NGS carried out by Exiqon. (**A**) Representative picture of cDNA library after adapter ligation and PCR amplification measured with Agilent Bioanalyzer2100. Bound and free adapter dimers are visible. Peak for sncRNAs is indicated. (**B**) Representative pictures of cDNA library separated on a 10% TBE Gel. Fragments corresponding to sncRNAs from approx. 18 to 36bp were cut (left: before cutting. Right: after cutting). (**C**) Representative picture of cDNA library after gel purification analyzed with Agilent Bioanalyzer2100 shows the sncRNA peak but no adapter fragments. (**D**) Representative pictures of average read Q-scores from data quality control after NGS. All data have a Q-score > 30 (red line), indicating more than 99.9% accuracy of base calling. (**E**) Blue bars show percentage of reads with the indicated score. (**F**) Read length distribution after adapter trimming reveals a prominent miRNA peak with 18-22 nt and few longer sequences of 30 – 50 nt belonging to other ncRNAs such as tRNAs, rRNAs, ect. (**G**) Total mapped reads of sequencing. Reads were annotated to miRBase20 and classified according to the following categories: 'not aligned', 'outmapped', 'genome-mapped',smallRNA' and 'miRNA'. (**H**) % of total mapped reads. Reads were annotated to miRBase20 and classified according to the following categories: 'not aligned', 'outmapped', 'genome-mapped', 'smallRNA' and 'miRNA'.

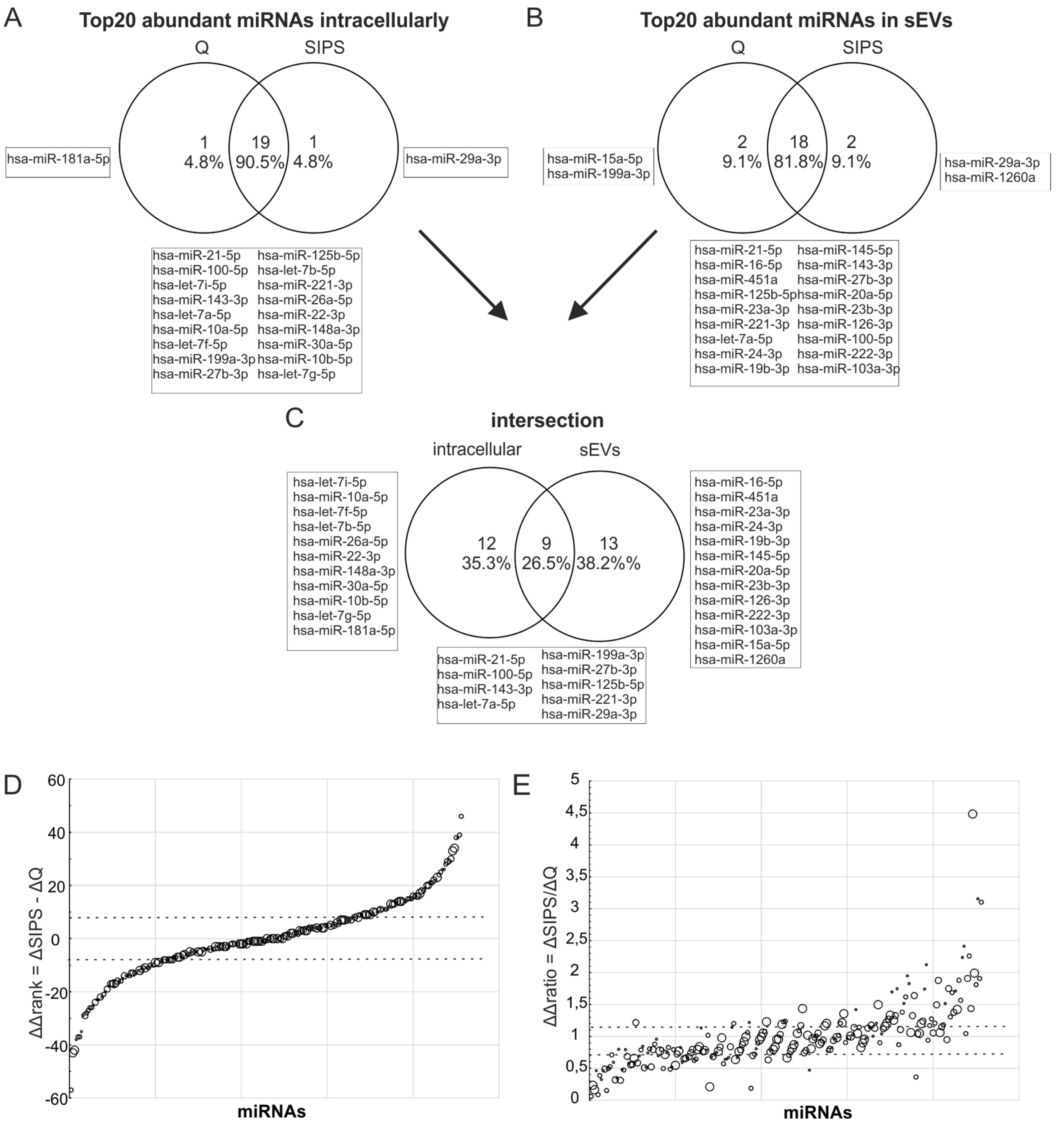

Figure S6. (**A**) Venn diagram of the top 20 abundantly transcribed miRNAs in cells from Q and SIPS cells, sorted by ranks, used to identify commonly transcribed miRNAs in HDF. (**B**) Venn diagram of the top 20 abundant sEV-miRNAs secreted from Q and SIPS cells, sorted by ranks, used to identify commonly secreted sEV-miRNAs of HDF. (**C**) Positively ('mirroring effect' of inside and outside) and negatively matching miRNAs are identified by building the intersection from A + B. 26.5% matching miRNAs were found. (**D**) Specifically senescence-associated secreted (high values) or retained (low values) miRNAs are identified by the rank method. ΔΔrank values were calculate from Δrank values derived from Q and SIPS separately. High ΔΔranks indicate 'secreted' and low ΔΔranks indicate 'retained'. Bubble size corresponds to the average expression value from the transformed Ct-values. Dotted lines represent the 25% and 75% percentiles, which defines the cut-off for specifically secreted and retained miRNAs in SIPS .ΔΔrank: 25%: 8.0; Median: -0.5; 75%: 9.0; (**E**) Specifically senescence-associated secreted (high value) or retained (low value) miRNAs are identified by the ratio method. Ratios between intracellular and vesicular values are calculated. By further calculating and normalizing ΔΔratios, specifically senescence-associated secreted (high values) or retained (low values) miRNAs are identified. Due to differences in units, it is not possible to set the threshold to 1. Results are sorted from smallest to largest. They are plotted in the same manner as it resulted after sorting of ΔΔratios and appear in a similar shape as in (D), indicating that we identified a similar set of miRNAs. High ΔΔranks indicate 'secreted', and low ΔΔranks indicate 'retained'. Bubble size corresponds to average expression value from transformed Ct-values. Dotted lines represent the 25% and 75% percentiles, which define the specifically secreted and retained miRNAs in SIPS ΔΔratio: 25%: 0.7099; Median: 0.927; 75%: 1.186.

Supplementary Methods

Annexin-V-PI staining

For staining of apoptotic cells, the Pacific Blue™ Annexin-V Kit (Biolegend, San Diego, CA, USA, 640918) was used. Cells and supernatants were harvested, pooled, centrifuged at 200 x g for 10 minutes (min) and pellets were washed twice with Annexin-V binding buffer (10 mM Hepes/NaOH pH 7.4, 140 mM NaCl, 5 mM $CaCl_2$). After centrifugation at 500 x g, the pellet was resuspended and incubated for 15 min in Annexin-V/PI staining solution (250 ng/mL propidium iodide PI, Sigma Aldrich GmbH, St Louis, MO, USA P4864, 200 ng/mL Pacific Blue, diluted in Annexin-V binding buffer). The analysis was performed on a Gallios flow cytometer (Beckman coulter, Brea, CA, USA) using an excitation wavelength of 488 nm and a 600 nm emission filter for detection of PI (FL-3) and an excitation of 405 nm and a 450/50nm emission filter for Pacific-Blue-Annexin (FL-9). Cells treated with 300 nM Staurosporin for 24 hours were used as a positive control. Flow cytometry data were analyzed with Kaluza software (Beckman Coulter, Brea, CA, USA, Version 1.2).

BrdU incorporation

In order to verify growth arrest, cells were incubated for 24 hours with 10 µM BrdU (Sigma Aldrich GmbH, St Louis, MO, USA, B5002). The cells were harvested by trypsinization, centrifuged at 170 x g for 5 min and the pellet was fixed with ice cold 70% ethanol for at least one hour at 4°C. Cells were permeabilized for 30 min with 2 M HCl and 1% Triton X-100 (Sigma Aldrich GmbH, St Louis, MO, USA, X100), followed by neutralization with 0.1 M Na-Borat, pH 8.5. Pellets were resuspended in TBS (0.5% Tween20, 1% BSA in 1 x PBS) containing anti-BrdU antibody 1:50 (BD Biosciences, USA, 347580) and incubated for 30 min. After washing with TBS and counterstaining with anti-mouse FITC-conjugated antibody 1:100 (Sigma Aldrich GmbH, St Louis, MO, USA F8264) for 30 minutes, the pellet was washed with TBS and resuspended in1 x PBS with 2.5 µg/ml PI (Sigma Aldrich GmbH, St Louis, MO, USA, P4864). For compensation, cells were stained with either PI or BrdU alone. The analysis was performed by flow cytometry (Gallios Beckman coulter, Brea, CA, USA), using an excitation wavelength of 488 nm and a 600 nm emission filter for detection of PI (FL-3) and a 535 nm filter for BrdU-FITC (FL-1). Proliferating cells were used as positive controls. Flow cytometry data were analyzed with Kaluza software (Beckman Coulter, Brea, CA, USA, Version 1.2).

Senescence associated (SA) ß-Gal staining

SIPS HDF and sub-confluent HDF at the middle of their replicative lifespan were stained according to the standard protocol described by Dimri et al. 1995 [1]. 15 pictures per well were taken at 100 x magnification and after randomization and blinding, SA-ß-Gal positive and negative cells were counted.

Nanoparticle tracking analysis

Experiments related to sEV Isolation were performed according to standards recommended from the international society for extracellular vesicles (ISEV) [2].

For determination of size and concentration of vesicles, the ZetaView® system (Particle Metrix, Meerbusch, Germany) was used. After calibrating the system with 110 nm polystyrene standard beads (Particle Metrix, Meerbusch, Germany), vesicles resuspended in 1000 µl after ultracentrifugation were diluted 1:200 in filtered 1 x PBS and 3 consecutive measurements were performed. Camera sensitivity was adjusted to fit the highest and lowest concentrated sample into the dynamic range and all samples were measured with the same dilution and settings. Settings: Gain 904, 98; Offset 0. Measurements were taken at two different camera positions and a total of ~$1x10^{10}$ particles/cm² were tracked, which corresponds to 150 – 400 counted particles per measurement. Particles secreted per cell were calculated using the cell number measured with an automated cell counter, Vi-CELL XR (Beckman Coulter, Brea, CA, USA). Categories of particle size determination was defined by the device. Categories below 15 nm, 15 nm, 45 nm, 105 nm, 135 nm, 165 nm, 195 nm and bigger than 225 nm are shown.

Electron microscopy

SEVs for Transmission Electron microscopy (TEM) were freshly prepared. Solutions used for the staining procedure were pre-filtered using 0.22 µm filter units (Millipore, Germany, SCGPU05RE). SEVs were adhered on Athene Old 300 mesh copper grids (Agar Scientific, Stansted, Essex, UK) and fixed with 1% glutaraldehyde. After washing three times with nuclease free water, vesicles were stained for 5 min with 2% phosphotungstic acid hydrate (Carl Roth, Karlsruhe, Germany). The grids were left to dry and the specimens were visualized using TEM (FEI Tecnai T20, FEI Eindhoven, Netherlands) operated at 160 kV.

Protein quantification, western blot and antibodies

Vesicles and corresponding cells were lysed in 1 x TNE buffer (2 x TNE: 100 mM Tris/HCl, pH 8.0, 300 mM

NaCl, 1 mM EDTA, 2 % Triton X-100) to quantify membrane markers of sEVs. Protein content of lysates was quantified with the Pierce® BCA Protein Assay Kit (Thermo Scientific, USA, 23227) according to manufacturer's recommendations and equal amounts of protein were loaded onto the gel (20 µg). For SDS page and subsequent western blotting, samples were resuspended in SDS loading dye (4 x SDS loading dye: 240 mM Tris/HCl, pH 6.8, 8% SDS, 40% glycerol, 0.05% bromophenolblue, 5% ß-Mercaptoethanol), sonicated and heated to 95°C. Then, samples were separated on a NuPAGE 4–12% Bis/Tris polyacrylamide gel (Invitrogen/Thermo Scientific, USA, 10472322) at 200V and proteins were transferred to a PVDF membrane (Biorad, Hercules, CA, USA, 170-4156) in a BioRad SemiDry Blotting System at 1.3A 25V for 7 minutes. Membranes were incubated with antibodies targeting TSG101 1:2000 (Abcam, ab125011) and GAPDH 1:1000 (pierce, MA5-15738). Proteins were detected using secondary antibodies for IRDye® 800CW Donkey anti-Rabbit IgG, 0.5 mg (LI-COR Biosciences, USA, 926-32213) and IRDye® 680RD Donkey anti-Mouse IgG, 0.5 mg (LI-COR Biosciences, USA, 926-68072) with a 1:10000 dilution using the Odysee (LI-COR Biosciences, USA) infrared image system. All antibodies were diluted in 3% milk-powder dissolved in 1 x PBS with 0.1% Tween-20 (Sigma Aldrich GmbH, St Louis, MO, USA, P2287).

Supplementary References

1. Dimri GP, Lee XH, Basile G, Acosta M, Scott C, Roskelley C, Medrano EE, Linskens M, Rubelj I, Pereirasmith O, Peacocke M, Campisi J. A Biomarker That Identifies Senescent Human-Cells in Culture and in Aging Skin in-Vivo. Proc Natl Acad Sci U S A. 1995; 92: 9363–7.

2. Hill AF, Pegtel DM, Lambertz U, Leonardi T, Driscoll LO, Pluchino S, Ter-ovanesyan D, Hoen ENMN-. ISEV position paper: extracellular vesicle RNA analysis and bioinformatics. 2013; 1: 1–8.

Supplementary Data Set

Please browse Full Text version to find the data related to this manuscript.

Supplementary Lists in one Excel spreadsheet 2017_Terlecki_HDF_H2O2_SIPS_Supplementary_Lists.

S1 S1_customized_QPCR_panel S2_Secreted_per_cell S3_pathway_miRNA_gene_interact S4__EV_composition_GlobalMean S5_Intracellular S6_Correlation_Top20 S7_details_correlation
